# Early L-T4 intervention improves fetal heart development in pregnant rats with subclinical hypothyroidism rats by activating BMP4/Smad4 signaling pathway

**DOI:** 10.1186/s12872-020-01646-3

**Published:** 2020-08-14

**Authors:** D. Xue, J. L. Sun, J. Yang

**Affiliations:** 1 Department of Cardiovascular Ultrasound, The First Hospital of China Medical University, No.115, Nanjing Road, HePing District, Shenyang, 110001 China; 2 Department of Cardiovascular Ultrasound, General Hospital of Northern Theater Command, Shenyang, China; 3 Department of Gynaecology and Obstetrics, General Hospital of Northern Theater Command, Shenyang, China

**Keywords:** Subclinical hypothyroidism, Cardiac development, Pregnant rats, L-T4, BMP4/Smad4 signaling pathway

## Abstract

**Background:**

It is unclear whether the offspring of subclinical hypothyroidism (SCH) pregnant rats still have abnormal cardiac development, and whether early intervention with L-T4 can improve the abnormality of these offspring. Therefore, the aim of this study was to investigate the effect of early L-T4 intervention on the heart development of offspring of SCH pregnant rats and its possible molecular mechanism.

**Methods:**

Eighty female Wistar rats were randomly divided into Sham group (placebo control), SCH group, LT4-E10 group (L-T4 treatment started on the 10th day of gestation), and LT4-E13 group (L-T4 treatment started on the 13th day of gestation). Each group was further divided into E16 (16th day of gestation), E18 (18th day of gestation), P5 (5th day postnatal day), and P10 (10th day postnatal day) subgroups. The levels of serum TT4 and TSH, the ratio of heart weight to body weight of offspring rats, the expression of metabolic enzymes, and the histopathology of cardiomyocytes were determined. To elucidate the effects of L-T4 on cardiac development of offspring of SCH pregnant rats, the expression levels of GATA4, Nkx2–5 and proteins involved in BMP4/Smad4 signaling pathway were detected by immunohistochemistry, real time quantitative polymerase chain reaction and Western blotting to elucidate the molecular mechanism of L-T4 regulating the heart development of the offspring of SCH pregnant rats.

**Results:**

Compared with Sham group, serum TSH was significantly increased in SCH pregnant rats. Moreover, early L-T4 intervention significantly reduced the levels of serum TSH. Compared with the offspring in the SCH group, early L-T4 intervention significantly increased the heart weight, heart weight to body weight ratio, the activities of succinate dehydrogenase (SDH), Na^+^/K^+^-ATPase and Ca^2+^-ATPase, but reduced myocardial cell shrinkage and nuclear staining, hyperemia/congestion and vacuolar degeneration. In addition, early L-T4 intervention not only significantly increased the mRNA and protein expression of Gata4 and Nkx2–5, but also increased the protein expression involved in BMP4/Smad4 signal pathway in myocardium of the offspring of SCH pregnant rats.

**Conclusions:**

Early L-T4 intervention can regulate the cardiac development of the offspring of SCH pregnant rats by activating BMP4/Smad4 signaling pathway and increasing the expression of Gata4 and Nkx2–5 proteins.

## Background

Subclinical hypothyroidism (SCH) is one of the most common thyroid diseases during pregnancy, affecting 2 to 3% of the global population. SCH was defined as increased serum thyroid-stimulating hormone (TSH) during pregnancy, but the level of free thyroxine (FT4) is normal [1]. Pregnancy has a great influence on the thyroid function of pregnant women. During pregnancy, human chorionic gonadotropin (HCG) weakly binds and stimulates TSH receptor and stimulates thyroid to produce more thyroxine. Meanwhile, the volume of thyroid increases by 10 to 40%, and the demand for thyroxine and iodine also increases by 50%, which will inevitably lead to hypothyroidism in the third trimester of pregnancy. Patients diagnosed with SCH before pregnancy have more severe symptoms of thyroid hormone deficiency during pregnancy [2]. It is known that poor control of SCH during pregnancy is related to pregnancy complications and growth retardation of offspring. In addition, women with hypothyroidism have an increased risk of hypertension and fetal death. These data indicate that there is a certain relationship between pregnant women with SCH and the risk of fetal disease [3].

Since the cardiovascular system is rich in thyroid receptor, it is relatively sensitive to the change of TSH levels [4]. Previous studies have showed that TSH levels above 7.0 mIU/L led to abnormalities in lipid metabolism, oxidative stress and endothelial function, thereby increasing the risk of atherosclerosis and congestive heart failure. In addition, patients with SCH under 65 years of age were associated with increased risk of coronary heart disease, heart failure, and cerebrovascular diseases. Patients with TSH levels above 10 mIU/L have a higher risk of heart failure with lower ejection fraction compared to participants with normal thyroid function [5]. The left ventricular diastolic dysfunction caused by SCH may be related to endothelial dysfunction, arterial stiffness and inflammation [6]. Another clinical study indicated that patients with SCH often had a slower heart rate and shorter corrected QT, which may be closely related to cardiac systolic dysfunction [7]. Longer P-wave duration, longer PR interval and decreased voltage were observed in patients with SCH. In addition, age and gender were known to be closely related to the occurrence of SCH [8]. However, it is still unclear the effect of pregnant women with SCH on fetal heart development and its possible molecular mechanism.

Although it is well known that women with hypothyroidism should increase the dose of levothyroxine during pregnancy, the exact timing and dose are not clear. A previous study found that women with hypothyroidism took administrated 150 μg/day of levothyroxine, a manufactured form of the thyroid hormone thyroxine (T4), during13 weeks of pregnancy, which can lead to an increase in TSH or a decrease in FT4 [9]. It has been confirmed that the demand for exogenous L-T4 dose of most pregnant women increase from 25 to 30% during the first 4 to 6 weeks of pregnancy and gradually increased within 16 to 20 weeks of pregnancy, and then stabilize until delivery [10]. It was reported that compared with healthy people, the left ventricular systolic and diastolic function of SCH patients was slightly affected. Moreover, L-T4 treatment reversed the change of left ventricular systolic and diastolic function of SCH patients, but has no significant effect on systolic pressure [11]. A similar study also found that for children with hypothyroidism and ventricular diastolic dysfunction, L-T4 treatment can significantly improve left ventricular dysfunction [12]. However, the effects of L-T4 on the fetal heart development of pregnant women with SCH and its possible mechanism are still unknown. Therefore, the aim of this preclinical study was to investigate the effect of L-T4 treatment on the fetal heart development in SCH pregnant rats and to explore its possible molecular mechanism.

## Methods

### Animals and groups

Eighty female Wistar rats, aged 6–8 weeks and weighing 180–200 g, were purchased from the Benxi Changsheng Biotechnology Co., Ltd. The animals were kept in the Experimental Animal Department of the Northern Theater General Hospital. The ambient temperature was maintained at 21–22 °C with 50–60% relative humidity. All rats were maintained under a 12:12-h light/dark cycle, with access to food and water ad libitum. Eighty rats were randomly divided into Sham group (placebo control), SCH group, LT4-E10 group (L-T4 treatment started on the 10th day of gestation), and LT4-E13 group (L-T4 treatment started on the 13th day of gestation). Each group was further divided into E16 (16th day of gestation), E18 (18th day of gestation), P5 (5th day postnatal day), and P10 (5th day postnatal day) subgroups. The animal experiment procedure was approved by the Animal Care and Use Committee of the General Hospital of Northern Theater Command, which complied with the national guidelines on the protection and use of laboratory animals.

### Establishment of the SCH rat model

Establishment of the SCH rat model refers to the previously literature [13].The rats were injected with 3% pentobarbital sodium (0.1 mL/100 g) and underwent thyroidectomy, while Sham group rats underwent sham thyroid surgery. The rats were fed normally for 4 w after operation, then the blood was collected from the retroorbital venous plexus, and serum TSH and TT4 were detected. When serum TSH levels were higher than that in Sham group, the TT4 levels were lower than that in Sham group, confirming the successful establishment of the SCH rat model. Four weeks after surgery, rats in the SCH group were injected subcutaneously with L-thyroxine (L-T4, Sigma, USA) 1.0 μg/100 g/day on the neck. Sham group rats were injected subcutaneously with physiological saline (50 μL/100 g/day) on the neck. Calcium lactate (0.1% w/v) was added to the drinking water for all rats after surgery. Nine days later, all rats were mated with normal male rats (male: female = 1: 2). The pregnant rats were then kept in single cages until delivery. The day of vaginal plus was confirmed by microscopic observation and designated as E0. Serum and tissue samples were collected at E16, E18, P5 and P10. At the end of the experiment, all rats were anesthetized with pentobarbital (50 mg/kg, intraperitoneal) and euthanized by thoracotomy and hearts removal.

### Measurement of TT4 and TSH

Blood samples obtained from the rats were immediately centrifuged at 13,000 g for 15 min and stored at − 80 °C for measurement of serum TT4 (^#^CEA452Ge, Youersheng, China) and TSH (^#^CEA463Ra, Youersheng, China) using a supersensitive chemiluminescence immunoassay.

### ATPase activity analysis

The ATPase activity was detected using an ultramicro Ca^2+^-ATPase kit and Na^+^/K^+^-ATPase kit (^#^A070, Nanjing Jiancheng Bioengineering Institute, China) according to the manufacturer’s instructions. The succinate dehydrogenase (SDH) activity was measured by kit(A022, Nanjing Jiancheng Bioengineering Institute, China). Protein content was measured with a Coomassie blue protein assay kit (^#^WLA004a, wanleibio, China). ATPase activity was expressed as mol Pi liberated per mg protein per hour (mol Pi/(mg prot·hr)).

### RNA isolation and quantitative real-time PCR

RNA was extracted from heart tissues from each group using TRIzol (Life Tech). First strand of cDNA was synthesized using total RNA and RT-PCR was carried out by TaqMan expression assays, and β-actin (^#^WL01845, wanleibio, China) was used as a internal reference. The sequences for Nkx2–5, Gata4, BMP4, Smad4 and GAPDH were performed through the ABI PRISM system. Primer sequences are shown: Bmp4 F 5′-ATCGTTACC TCAAGGGAGTGGA-3′; Bmp4 R 5′-ATCGTTACCTCAAGGGAGTGGA-3′; Samd4 F 5′-CGTTCACGAGGCATTTAC-3′; Samd4 R 5′-GGGAGGGAGTTGGACTG-3′; Nkx2–5 F 5′-TGGACAAAGCCGAGACAGAC-3′; Nkx2–5 R 5′-TCAGCGGGCGACAGGTA-3′; Gata4 F 5′-AAACGGAAGCCCAAGAAT-3′; Gata4 R 5′-GCTGCTGTGCCCATA GTGAG-3′; β-actin F 5′-GGAGATTACTGCCCTGGCTCCTAGC-3′; β-actin R 5′-GGCCGGACTCATCGTACTCCTGCTT. Reactions were performed in a total volume of 20 μL and gene expression was determined by SYBR Premix Ex Taq TM II (TaKaRa Biotechnology Co., Ltd.) in accordance with the manufacturer’s instructions. Reactions began with a 10 s hot activation of Taq polymerase at 95 °C, followed by 40–45 cycles of amplification in three steps (denaturation at 95 °C for 5 s, 30 s annealing at 60 °C and 30 s extension at 72 °C). The mRNA expression was measured as a ratio to β-actin.

### Immunohistochemistry staining

Immunohistochemistry staining was performed to localize Nkx2–5 (1:500, ^#^A12688, wanleibio, China), Gata4 (1:1000, ^#^WL01293, wanleibio, China), BMP4 (1:500, ^#^WL02806, wanleibio, China) and Smad4 (1:300, ^#^WL02049, wanleibio, China). Heart tissues were routinely embedded in paraffin, and sectioned at a 3–5 μ m thickness. All sections were dewaxed, and incubated with 0.3% H2O2 for 10 min. The antigen was repaired by heating 0.03 M citrate buffer (pH 6.0) for 40 min at 95 °C, and then incubated with 5% goat serum albumin for 20 min to block the non-specific binding sites. All sections were incubated with primary antibody overnight at 4 °C in a wet box and stained with sheep anti-rabbit IgG-HRP secondary antibodies (Abcam, UK). The sections were stained using 3,3-diaminobenzidine (DAB) (Sigma, USA) and counterstained with hematoxylin solution. Tissue sections without primary antibodies were the negative controls. Finally, samples were observed and photographed under a microscope.

### Western blotting

Western blotting was performed as described previously [14]. Heart samples were lysed in complete RIPA buffer (10 mM Tris-HCl pH 7.4, 150 mM NaCl, 1% NP40, 0.1% sodium dodecyl sulfate, 1 mM phenylmethylsulfonyl fluoride and 1× protease inhibitor cocktail [Roche]) and homogenized using a Sonic Dismembrator 100. The concentration of tissue protein was detected using a BCA protein concentration test kit(^#^WLA004, wanleibio, China), and equal amounts of soluble protein were separated on 10% polyacrylamide gels, transferred onto a nitrocellulose membrane, and followed by routine western blot analysis. Primary antibody: Nkx2–5 (1:500, ^#^A12688, wanleibio, China), Gata4 (1:1000, ^#^WL01293, wanleibio, China), BMP4 (1:500, ^#^WL02806, wanleibio, China) and Smad4 (1:300, ^#^WL02049, wanleibio, China). Proteins were visualized using a ClarityTM Western ECL Substrate (^#^WLA003; wanleibio, China) and a Tanon 5200 Full automatic chemiluminescence image analysis system (Tanon Science and Technology Co., Ltd., Shanghai, China).

### Statistical analysis

Statistical analysis was performed using SPSS 20.0 statistical software (IBM Corp., Armonk, NY, USA). Variables are expressed as the means ± standard error of the mean (SEM). Data were analyzed using t-tests and one-way ANOVA. Statistical significance was reached at a two-sided *p* < 0.05.

## Results

### Effects of L-T4 on serum TSH and TT4 expression in the offspring of SCH pregnant rats

**Fig. 1:**
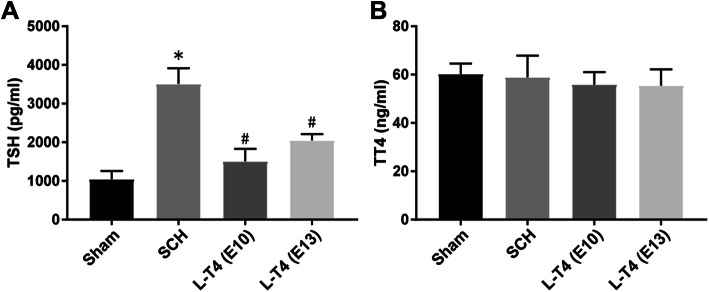
Effects of L-T4 treatment on the expression levels of serum TSH and TT4 in the offspring of SCH pregnant rats. **a** TSH expression level of pregnant rats in the Sham, SCH, LT4-E10 (L-T4 treatment started on the 10th day of gestation), and LT4-E13 (L-T4 treatment started on the 13th day of gestation) groups. There were 20 rats in each group. **b** TT4 expression level of pregnant rats in each group. ^*^p < 0.05 vs Sham group; ^#^*p* < 0.05 vs SCH group

In order to evaluate the thyroid function of SCH pregnant rats, the expression levels of serum TT4 and TSH of all pregnant rats were measured. The serum TSH level in SCH group was significantly higher than that in the Sham group (Fig. 1a, *p* < 0.05). There was no significant difference in TT4 expression level between the two groups, confirming the successful establishment of the SCH rat model (Fig. 1b). Interestingly, in the LT4-E10 and LT4-E13 groups, L-T4 treatment significantly reduced serum TSH expression level in SCH pregnant rats (Fig. 1a, compared to SCH group, *p* < 0.05). Moreover, there was no significant difference in the TT4 expression level between SCH, LT4-E10 and LT4-E13 groups. The results indicated that L-T4 treatment would affect the serum TSH expression levels in SCH pregnant rats, but did not change the expression level of TT4.

### L-T4 treatment promoted cardiac development in the offspring of SCH pregnant rats

**Fig. 2:**
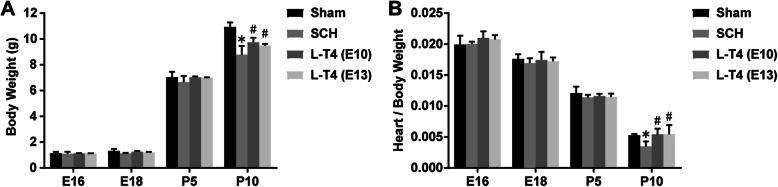
L-T4 promotes cardiac development in the offspring of SCH pregnant rats. **a** Body weight of the offspring of SCH pregnant rats. **b** The ratio of heart weight to body weight (Heart/Body Weight) of the offspring of SCH pregnant rats. Results are expressed as the mean ± SEM (*n* = 5 per group). ^*^p < 0.05 vs Sham group; ^#^*p* < 0.05 vs SCH group

To explore the effect of L-T4 on the heart development of the offspring of SCH pregnant rats, the heart weight and the ratio of heart weight to body (heart/body) weight were examined. The results showed that the heart weight and the heart/body weight of the offspring in the SCH group were significantly lower than that in the Sham group. Furthermore, in the LT4-E10 and LT4-E13 groups, L-T4 treatment significantly increased the heart weight and heart/body weight of the offspring of SCH pregnant rats (Fig. 2, compared to SCH group, *p* < 0.05). The results suggested that early administration of L-T4 can promote the heart development of the offspring of SCH pregnant rats.

### L-T4 treatment improved the metabolic function of cardiomyocytes in the offspring of SCH pregnant rats

**Fig. 3:**
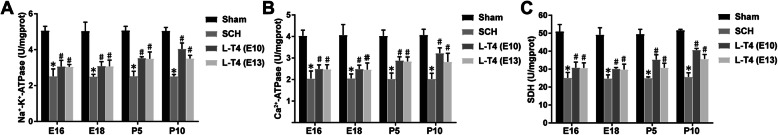
L-T4 treatment improved the metabolic function of cardiomyocytes in the offspring of SCH pregnant rats. (**a**) Na^+^/K^+^-ATPase activity (**b**) Ca^2+^-ATPase activity (**c**) SDH activity. All experiments were repeated at least three times. Data were expressed as the mean ± SEM (*n* = 5 per group). *p < 0.05 vs Sham group; ^#^*p* < 0.05 vs SCH group

To clarify the effects of L-T4 on the metabolic ability of cardiomyocytes of the offspring of SCH pregnant rats, the activities of SDH, Na^+^/K^+^-ATPase and Ca^2+^-ATPase were detected. The results showed that compared to Sham group, the activities of SDH, Na^+^/K^+^-ATPase and Ca^2+^-ATPase of the offspring were significantly decreased in the SCH group at E16, E18, P5 and P10. In the LT4-E10 and LT4-E13 groups, L-T4 treatment significantly increased the activities of SDH, Na+/K + -ATP and Ca2 + −ATP (Fig. 3, compared to SCH group, *p* < 0.05). The results suggested that L-T4 treatment regulated the cardiac development of the offspring of SCH pregnant rats by increasing the metabolic function of the cardiomyocytes.

### L-T4 treatment attenuated cardiac tissue injury of the offspring of SCH pregnant rats

**Fig. 4:**
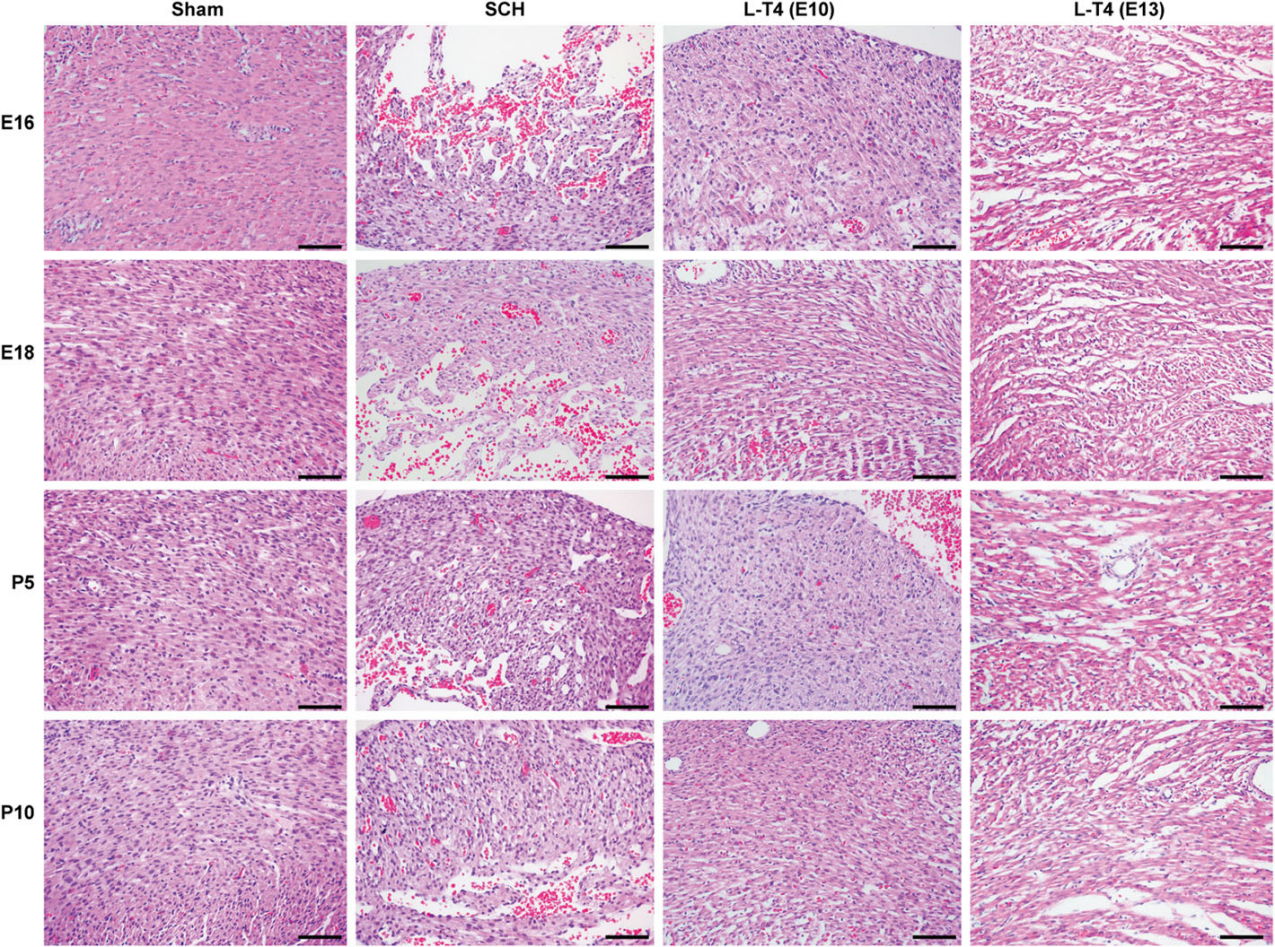
L-T4 attenuated cardiac tissue injury of the offspring of SCH pregnant rats. Representative histopathological images of each group (HE staining, scale bar = 50 μm)

The results of histopathological staining showed that the cardiomyocytes of the offspring in the Sham group were orderly arranged, and the structure of cardiomyocytes was clear and intact (Fig. 4). However, the cardiomyocytes of the offspring in the SCH group showed wrinkled, hyperchromatic and loosely arranged structures, with obvious hyperemia, congestion and vacuolar degeneration cell shrinkage, hyperchromatic nuclei, local hyperemia/congestion and vacuolar degeneration. Moreover, in the LT4-E10 and LT4-E13 groups, the degree of cardiomyocyte degeneration, inter-tissue congestion and vacuolar degeneration gradually improved after L-T4 treatment. The results suggested that L-T4 treatment during pregnancy can improve the pathological changes of the heart tissue of the offspring of SCH pregnant rats.

### L-T4 promoted the expression of cardiac development proteins in the offspring of SCH pregnant rats

**Fig. 5:**
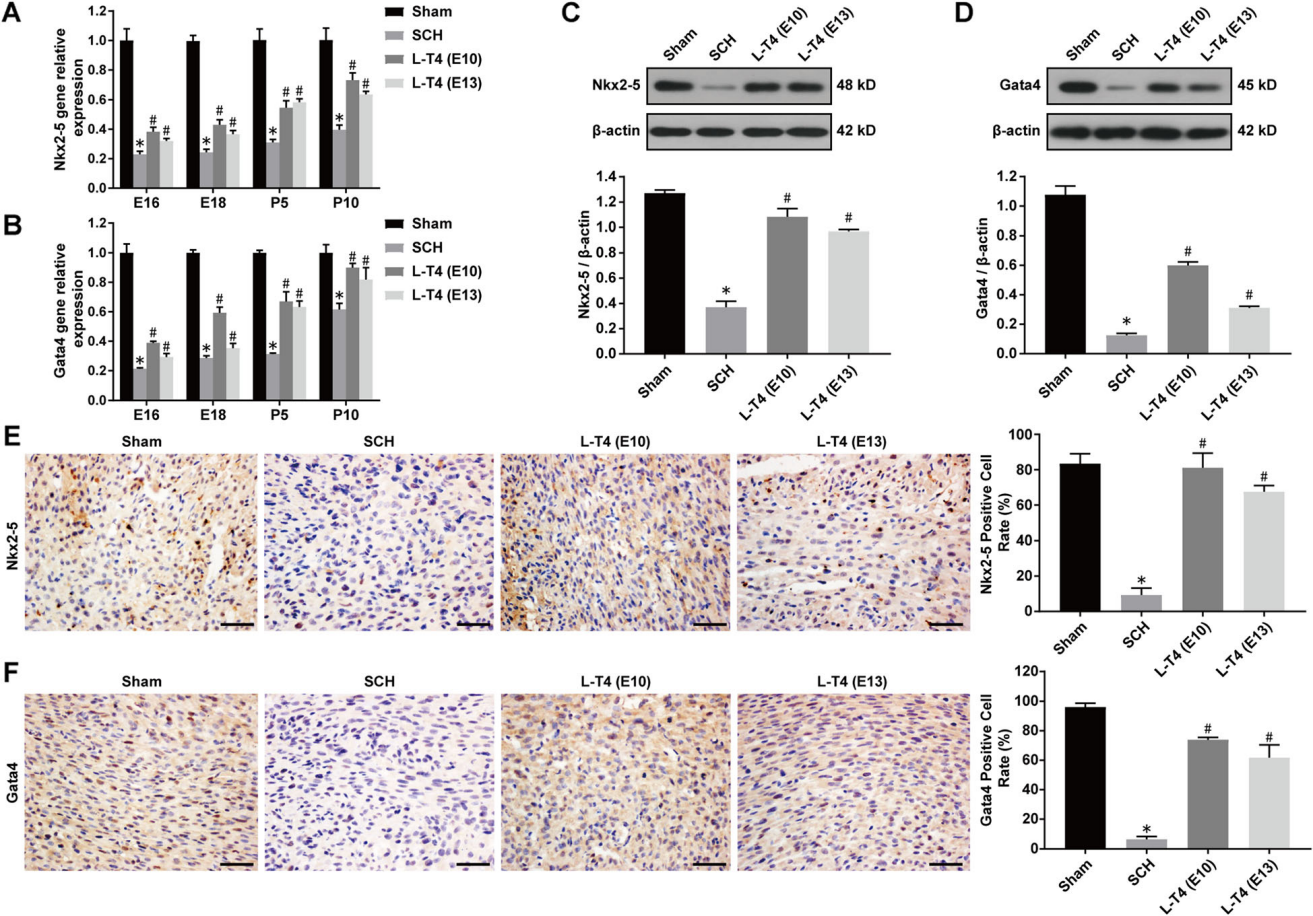
L-T4 treatment promoted the expression of cardiac development proteins in the offspring of SCH pregnant rats. **a**-**b** Expression levels of Gata4 and Nkx2–5 mRNA in the offspring of SCH pregnant rats; **c**-**d** Representative Western blot images and quantitative analysis of Gata4 and Nkx2–5 expression levels; **e**-**f** Representative immunohistochemical images of Gata4 and Nkx2–5 expression and quantitative analysis of Gata4- and Nkx2–5-positive cells in each group (Scale bar = 50 μm). All experiments were repeated at least three times. Data were expressed as the mean ± SEM (*n* = 5 per group). *p < 0.05 vs Sham group; ^#^*p* < 0.05 vs SCH group

Real-time quantitative PCR, immunohistochemistry and Western blotting were used to detect the expression of mRNAs and proteins related to cardiac development and differentiation of the offspring of SCH pregnant rats. The results showed that the expression levels of Nkx2–5 and Gata4 mRNA of the offspring in the SCH group was significantly lower than that in the Sham group. Furthermore, in the LT4-E10 and LT4-E13 groups, L-T4 treatment significantly increased the expression levels of Nkx2–5 and Gata4 mRNA in the myocardium of the offspring (Fig. 5a-b, compared to SCH group, *p* < 0.05). Western blotting analysis further confirmed the results that the expression levels of Gata4 and Nkx2–5 protein in the myocardium of the offspring in the SCH group was significantly lower than that in the Sham group, while L-T4 treatment further significantly increased the expression levels of Gata4 and Nkx2–5 protein in the myocardium of the offspring in the LT4-E10 and LT4-E13 groups (Fig. 5c-d, compared to SCH group, *p* < 0.05). Similarly, the results of immunohistochemical staining showed that Gata4 and Nkx2–5 proteins were mainly expressed in the nucleus of heart cells. Compared to Sham group, the number of Gata4- and Nkx2–5-positive cells of the offspring in the SCH group were significantly reduced. Moreover, L-T4 treatment significantly increased the number of Gata4- and Nkx2–5-positive cells of the offspring in the LT4-E10 and LT4-E13 groups (Fig. 5e-f, compared to SCH group, *p* < 0.05). The results suggested that L-T4 treatment can regulate the cardiac development of the offspring of SCH pregnant rats by increasing the expression of Gata4 and Nkx2–5 proteins.

### L-T4 treatment increased the expression of BMP4/Smad4 proteins in the offspring of SCH pregnant rats

**Fig. 6:**
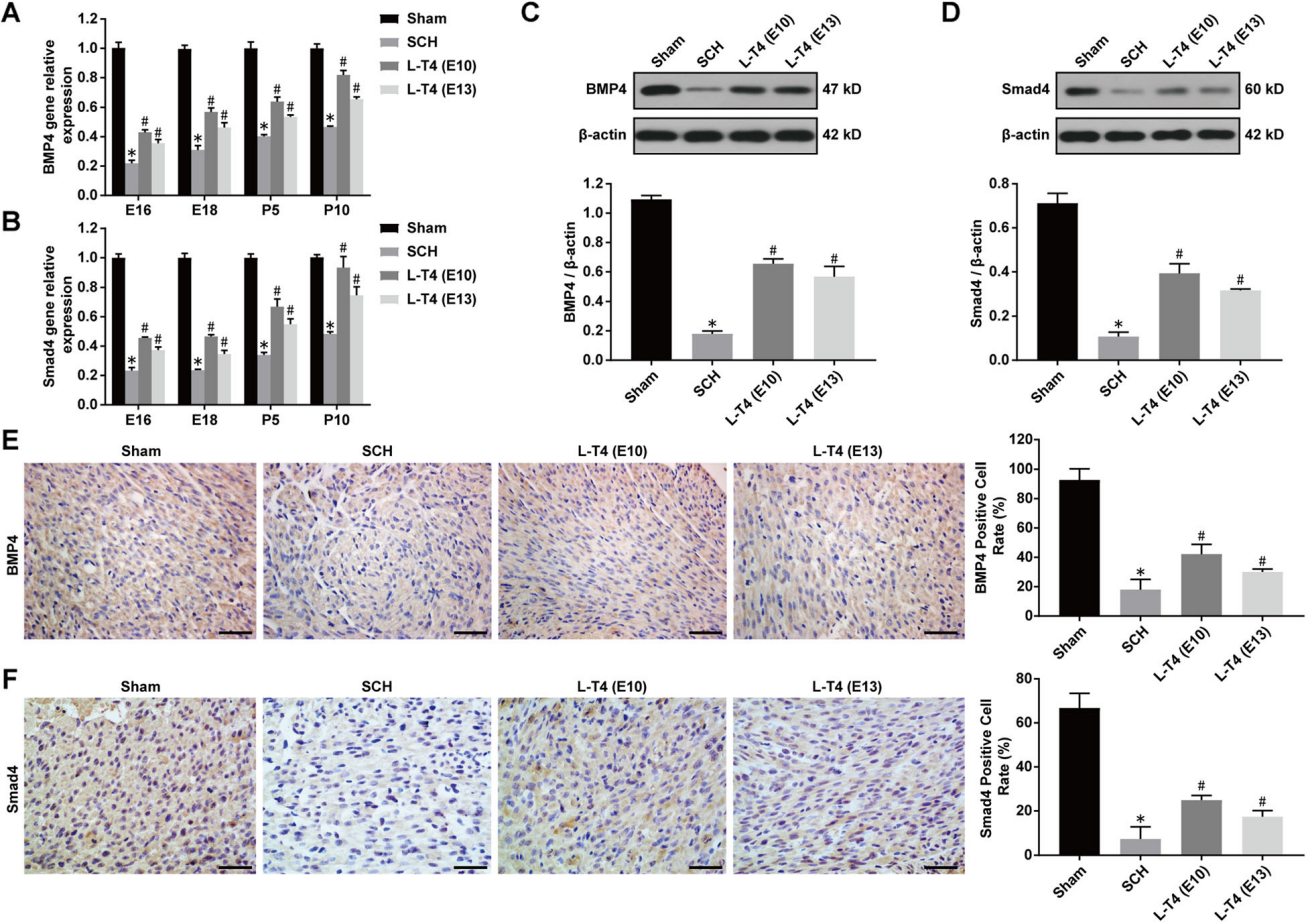
L-T4 treatment increased the expression of BMP4/Smad4 protein in the offspring of SCH pregnant rats. **a**-**b** Expression level of BMP4 and Smad4 mRNA in the offspring of SCH pregnant rats; **c**-**d** Representative Western blot images and quantitative analysis of BMP4 and Smad4 intensity in each group; **e**-**f**) Representative immunohistochemical images of BMP4 and Smad4 expression and quantitative analysis of BMP4- and Smad4- positive cells in each group (Scale bar = 50 μm). All experiments were repeated at least three times. Data were expressed as the mean ± SEM (*n* = 5 per group). ^*^p < 0.05 vs Sham group; ^#^*p* < 0.05 vs SCH group

Real-time quantitative PCR, immunohistochemistry and Western blotting were used to detect the BMP4 and Smad4 mRNA and protein levels of the offspring’s myocardium in SCH pregnant rats. The results showed that the mRNA levels of BMP4 and Smad4 in myocardial tissues of the offspring in the SCH group were significantly lower than that in the Sham group. Furthermore, L-T4 treatment significantly increased the expression of BMP4 and Smad4 mRNA in myocardial tissues of the offspring in the LT4-E10 and LT4-E13 groups (Fig. 6a-b, compared to SCH group, *p* < 0.05). The results of Western blotting analysis showed that the levels of BMP4 and Smad4 proteins in myocardial tissues of the offspring in the SCH groups were significantly lower than that in the Sham group, while L-T4 treatment significantly increased the expression of BMP4 and Smad4 proteins in both LT4-E10 and LT4-E13 groups (Fig. 6c-d, *p* < 0.05). Immunohistochemical staining showed that BMP4 and Smad4 were mainly located in the cytoplasm and nucleus of myocardial cells. The number of BMP4- and Smad4-positive myocardial cells in the offspring in SCH group was significantly lower than that in the Sham group. Similarly, L-T4 treatment significantly increased the number of BMP4- and Smad4-positive myocardial cells in the LT4-E10 and LT4-E13 groups (Fig. 6e-f, compared to SCH group, *p* < 0.05). The results suggest that L-T4 regulates the cardiac development of the offspring in SCH pregnant rats by activating BMP4/ Smad4 signal pathway.

## Discussion

Some studies have shown that the offspring of SCH pregnant rats were often accompanied by neurodevelopmental abnormalities, and early intervention with L-T4 can alleviate the neurodevelopmental abnormalities. However, it is not clear whether the offspring of SCH pregnant rats have cardiac developmental abnormalities, and whether L-T4 early intervention can improve the abnormalities of the offspring in SCH pregnant rats. The results of this study revealed that L-T4 treatment significantly decreased the serum TSH expression level in SCH pregnant rats, increased the heart weight and heart/body weight ratio of the offspring in SCH pregnant rats, improved the metabolic function of myocardial cells, and alleviated the pathological changes of myocardial tissues. In addition, L-T4 significantly increased the mRNA and proteins expression of Gata4, Nkx2–5, BMP4, and Smad4 of the offspring in SCH pregnant rats. The results suggest that L-T4 early intervention regulates the cardiac development of the offspring in SCH pregnant rats by activating BMP4/Smad4 signaling pathway, and then increasing the expression of Gata4 and Nkx2–5 proteins.

When pregnant women have hypothyroidism, abnormal thyroid hormone levels can severely affect the development of neuromotor, auditory, cardiovascular, and respiratory systems [15]. Thyroid hormones can promote the transformation of fetal cardiomyocytes from proliferation to hypertrophy and differentiation during full-term and early pregnancy [16]. Since the thyroid function of pregnant women affects the growth and maturation of fetal organs, the birth weight of the baby can indirectly reflect the level of thyroid function of pregnant women [17]. When the concentration of fetal thyroid hormone is kept within a relatively narrow range, it will not affect the normal development of the heart [18].Similarly, the concentration of T4 in umbilical cord blood was positively correlated with the birth weight and height of the baby, because thyroid hormones can provide nutrition and oxygen signals to the fetus in the uterus to regulate fetal growth [19]. Therefore, fetal thyroid hormone is essential for the increase of fetal weight and differentiation of specific cell types at the critical stage of development. Moreover, SCH caused growth retardation of fetal rats in the uterus, and even led to abortion or premature delivery [13] and permanent neurological defects of the offspring [20, 21]. In this study, we found that L-T4 early intervention not only significantly decreased the serum TSH levels in the SCH pregnant rats, but also increased the heart weight and heart/body weight ratio of the offspring, improved the metabolic function of myocardial cells of the offspring, and alleviated the pathological changes of myocardial tissues of the offspring. It was reported that thyroid hormone deficiency affected the expression profiles of myomiR network in the heart of fetal rats and the expression of downstream targeting genes, which in turn led to increased expression of b-MHC and related cardiac dysfunction in adulthood [22]. An infant with ectopic thyroid tissue was found to have sinus bradycardia associated with congenital hypothyroidism [23]. Another study found that SCH was associated with decreased cardiac output in patients with impaired vascular smooth muscle relaxation and reduced nitric oxide level. These changes were related to the decreased expression of sarcoplasmic reticulum Ca^2+^-ATPase and the inhibition of expression of ATP enzyme phosphoprotein [24]. Low circulating levels of thyroid hormone inhibited the activity of sarcoplasmic reticulum calcium ATP enzyme, which controlled the contraction and relaxation cycle through ATPase, leading to myocardial stiffness and eventually left ventricular diastolic dysfunction [25]. In addition, L-T4 treatment significantly improved low density lipoprotein, total cholesterol, hypertension, diastolic dysfunction and delayed arteriosclerosis in patients with SCH [26]. These results suggest that L-T4 treatment can improve the cardiac development of the offspring in SCH pregnant rats, which was related to increasing the heart weight, improving the metabolic function of the cardiomyocytes and alleviating the pathological changes of cardiomyocytes.

During the early development of embryonic heart, many genes such as bone morphogenetic proteins (BMPs), fibroblast growth factor and activin/nodule are involved in heart development and differentiation [27, 28]. BMP signaling pathway played a central role in the induction of mesoderm and heart development. The BMP ligand binds to type II receptor and then activates type I receptor to phosphorylate Smad1, Smad5 or Smad8 signal transduction pathway regulated by BMP receptor [29]. When BMP is released from the receptor complex, the phosphorylated R-Smad binds to the common Smad4 to form a trimeric complex composed of two R-Smad and Smad4, and then induces the transcription of downstream genes [30]. BMP4 knockout Fetal mice died within 9.5 days after birth, and most of them had little or no mesoderm differentiation [31]. BMP4 can induce the expression of Nkx2–5 and Gata4 in cardiac progenitor cells, which is necessary for cardiac development and differentiation [32]. Gata4 is one of the key transcription factors in fetal heart development. Abnormal expression of Gata4 can cause various fetal heart malformations, such as septal defect, tetralogy of Fallot, myocardial trabecular dysplasia, and valve malformation [33–35]. In this study, our results showed that the mRNA and protein expression levels of Gata4, Nkx2–5, BMP4, and Smad4 of the offspring were significantly decreased in SCH pregnant rats. Remarkably, L-T4 early intervention not only significantly increased the mRNA and protein levels of Gata4 and Nkx2–5, but also activated the BMP4/Smad4 signaling pathway in the myocardium of the offspring of SCH pregnant rats. It was reported that exposure of pregnant rats to di (2-ethylhexyl) phthalate caused cardiac malformation in the offspring, which may be related to the inhibition of cardiac Gata4/mef2c/ch expression [36]. Similarly, the potential toxicity of PM2.5 to fetal heart tissue was related to the down-regulation of transcriptional factors Gata4 and Nkx2–5, which were functionally associated with to embryonic heart development and abnormalities of fetal heart structure and function [37]. Moreover, exposure of pregnant rats to PM2.5 aggravated cardiovascular dysplasia caused by homocysteine in their offspring [38]. These results indicate that early treatment of L-T4 can significantly improve the myocardial development of the offspring of SCH pregnant rats, which may be related to the activation of BMP4/Smad4 and the regulation of Gata4 and Nkx2–5 protein expression.

## Conclusion

In conclusion, early administration of L-T4 can regulate the cardiac development of the offspring of SCH pregnant rats by activating BMP4/Smad4 signal pathway, and then increasing the expression of Gata4 and Nkx2–5.

## Supplementary information

**Additional file 1.**

## Data Availability

The datasets generated and analysed during the current study are not publicly available due the principle of confidentiality of funding, but are available from the corresponding author on reasonable request.

## Abbreviations

SCH: Subclinical hypothyroidism
PPARγ: Peroxisome proliferator-activated receptor gamma
LT4-E10 group: L-T4 treatment started on the 10th day of gestation
SDH: Succinate dehydrogenase
T4: Thyroid hormone thyroxine
TSH: Thyroid-stimulating hormone
BMPs: Bone morphogenetic proteins
