# Supplementary figures and images for: Early L-T4 intervention improves fetal heart development in pregnant rats with subclinical hypothyroidism rats by activating BMP4/Smad4 signaling pathway
Source: BMC Cardiovasc Disord. 2020 Aug 14;20:369. doi: 10.1186/s12872-020-01646-3 (PMC7427857; doi:10.1186/s12872-020-01646-3)

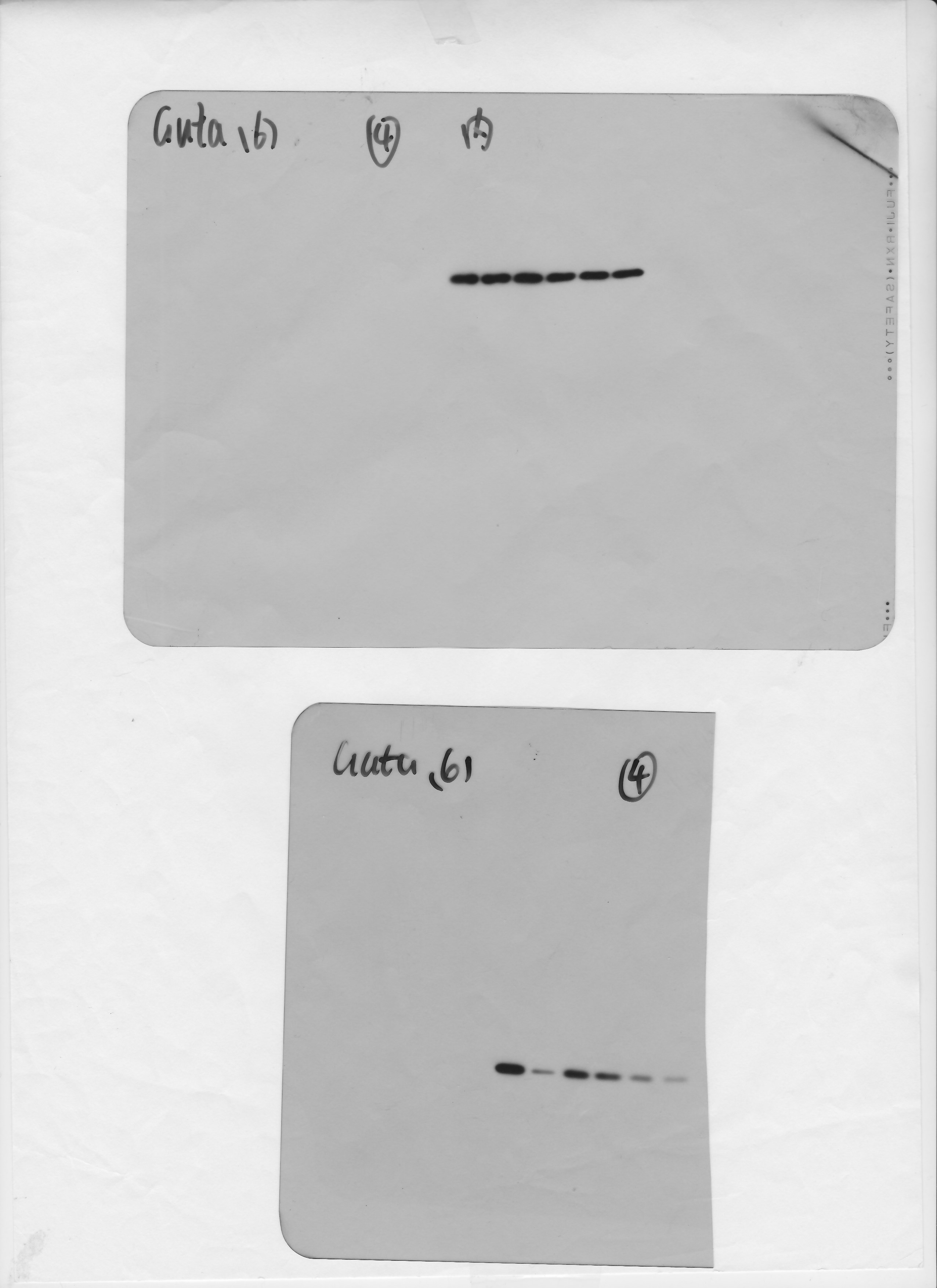

Supplement: Supplementary file 1 — Additional file 1. [file 12872_2020_1646_MOESM1_ESM.zip › 1.jpg]

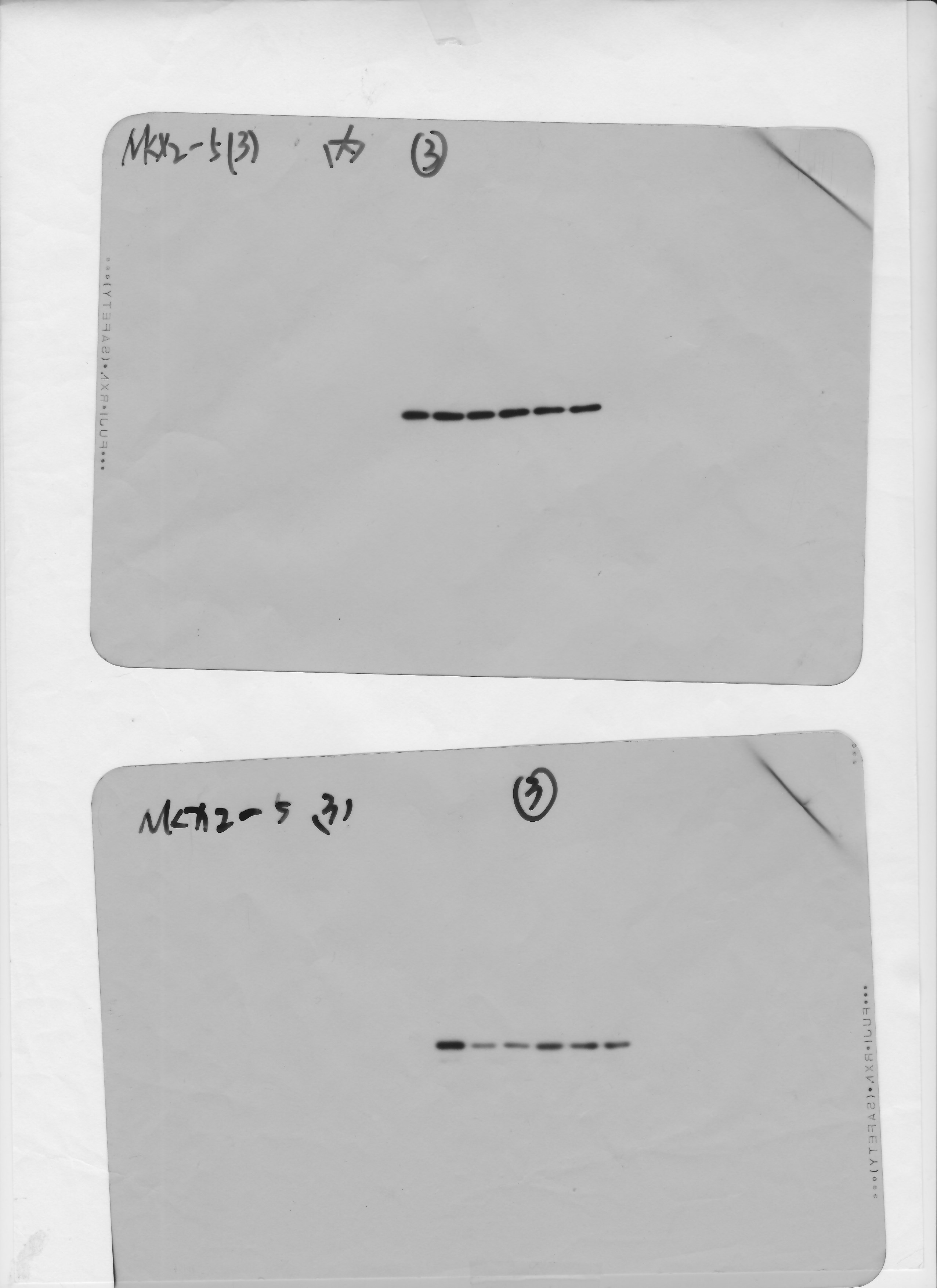

Supplement: Supplementary file 1 — Additional file 1. [file 12872_2020_1646_MOESM1_ESM.zip › 10R3.jpg]

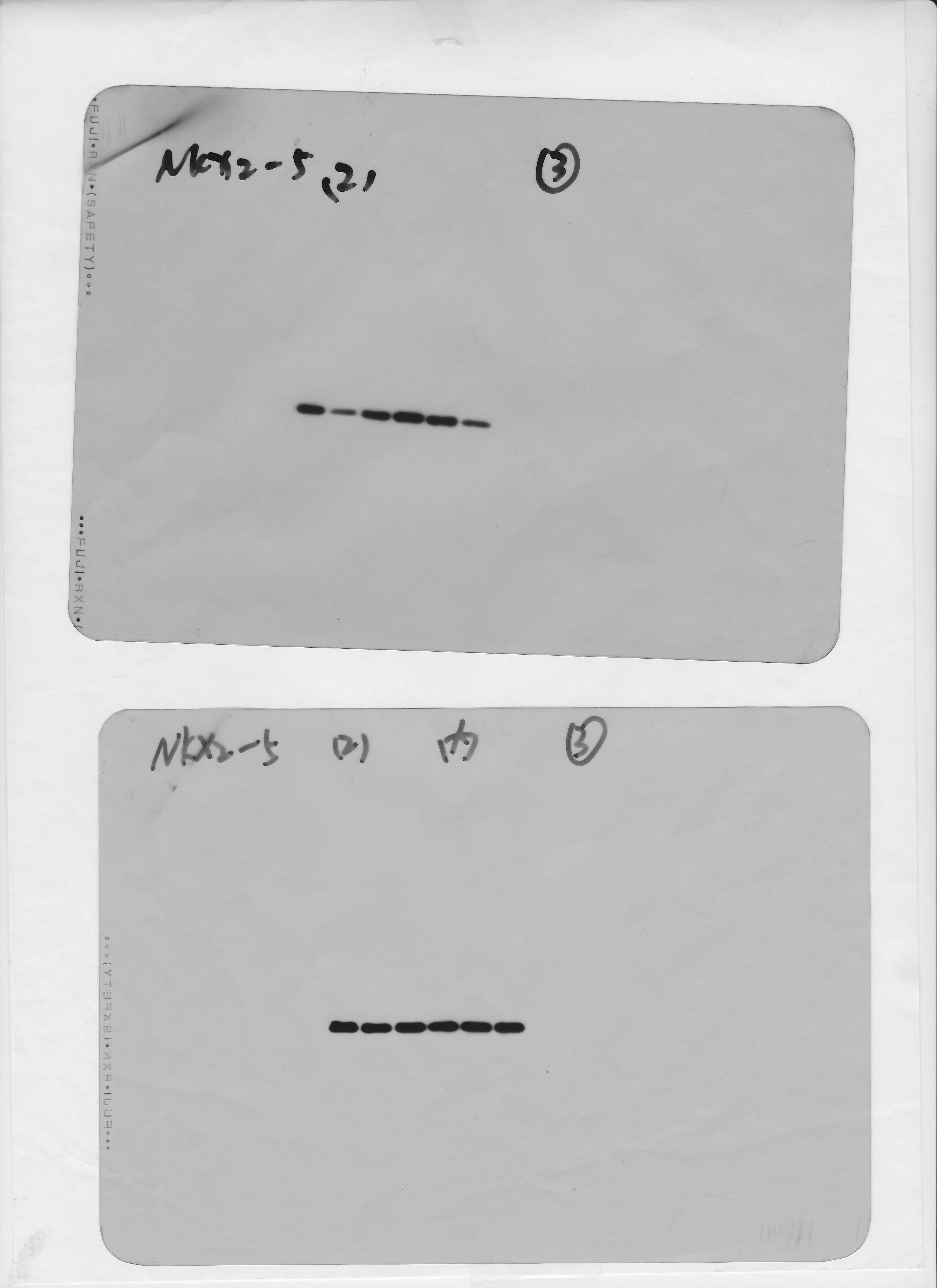

Supplement: Supplementary file 1 — Additional file 1. [file 12872_2020_1646_MOESM1_ESM.zip › 11R3.jpg]

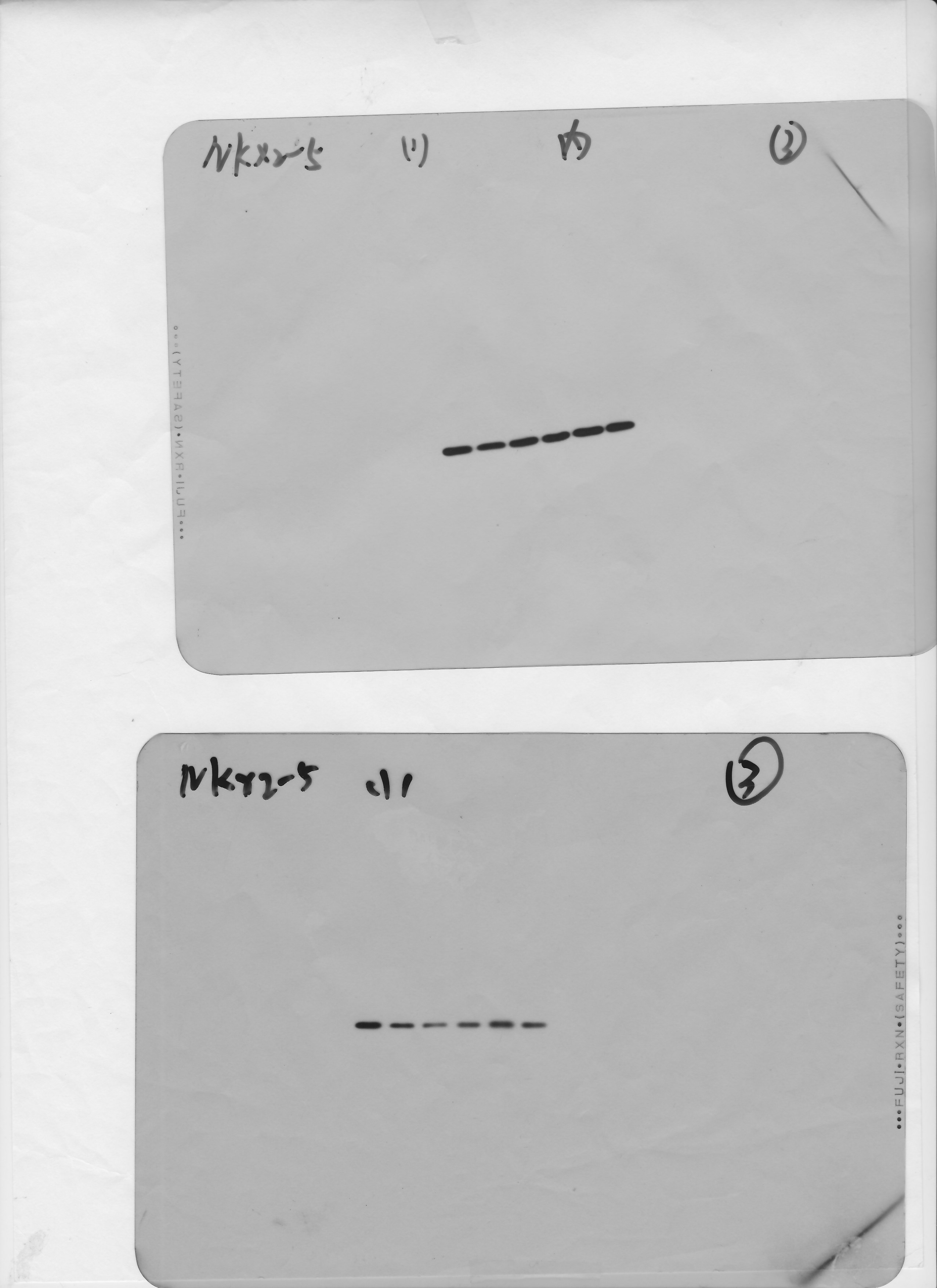

Supplement: Supplementary file 1 — Additional file 1. [file 12872_2020_1646_MOESM1_ESM.zip › 12R3.jpg]

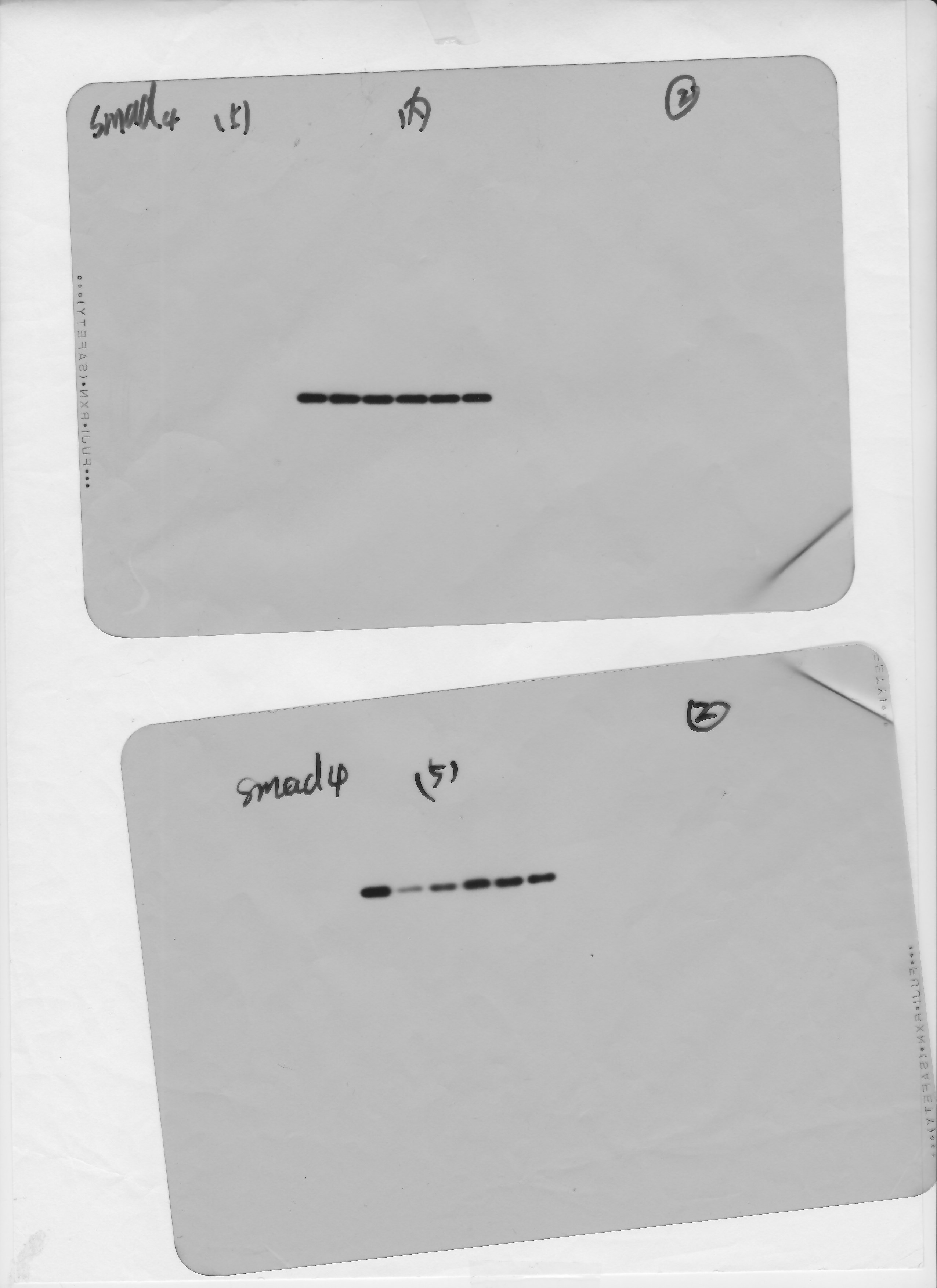

Supplement: Supplementary file 1 — Additional file 1. [file 12872_2020_1646_MOESM1_ESM.zip › 14R3.jpg]

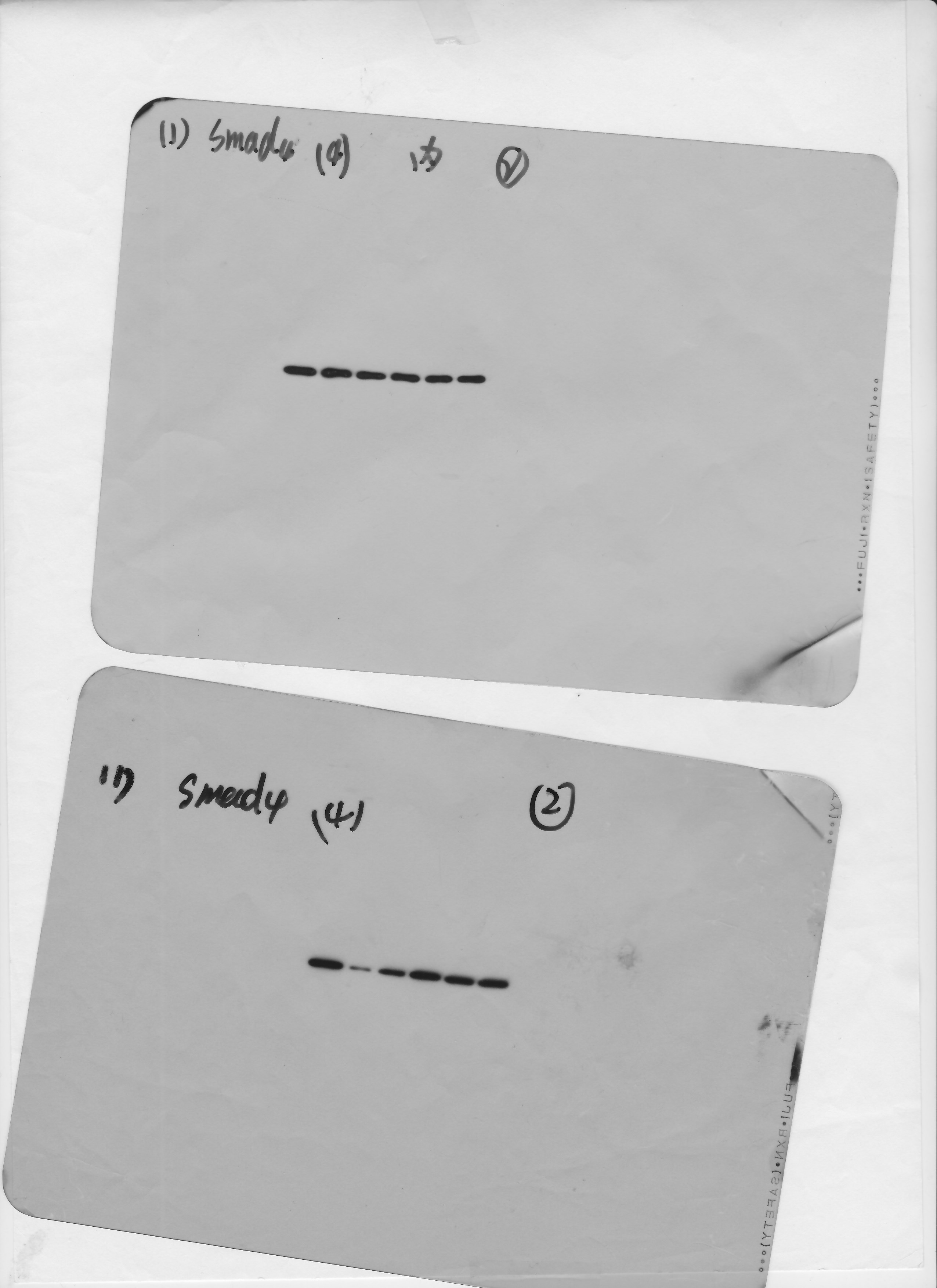

Supplement: Supplementary file 1 — Additional file 1. [file 12872_2020_1646_MOESM1_ESM.zip › 15R3.jpg]

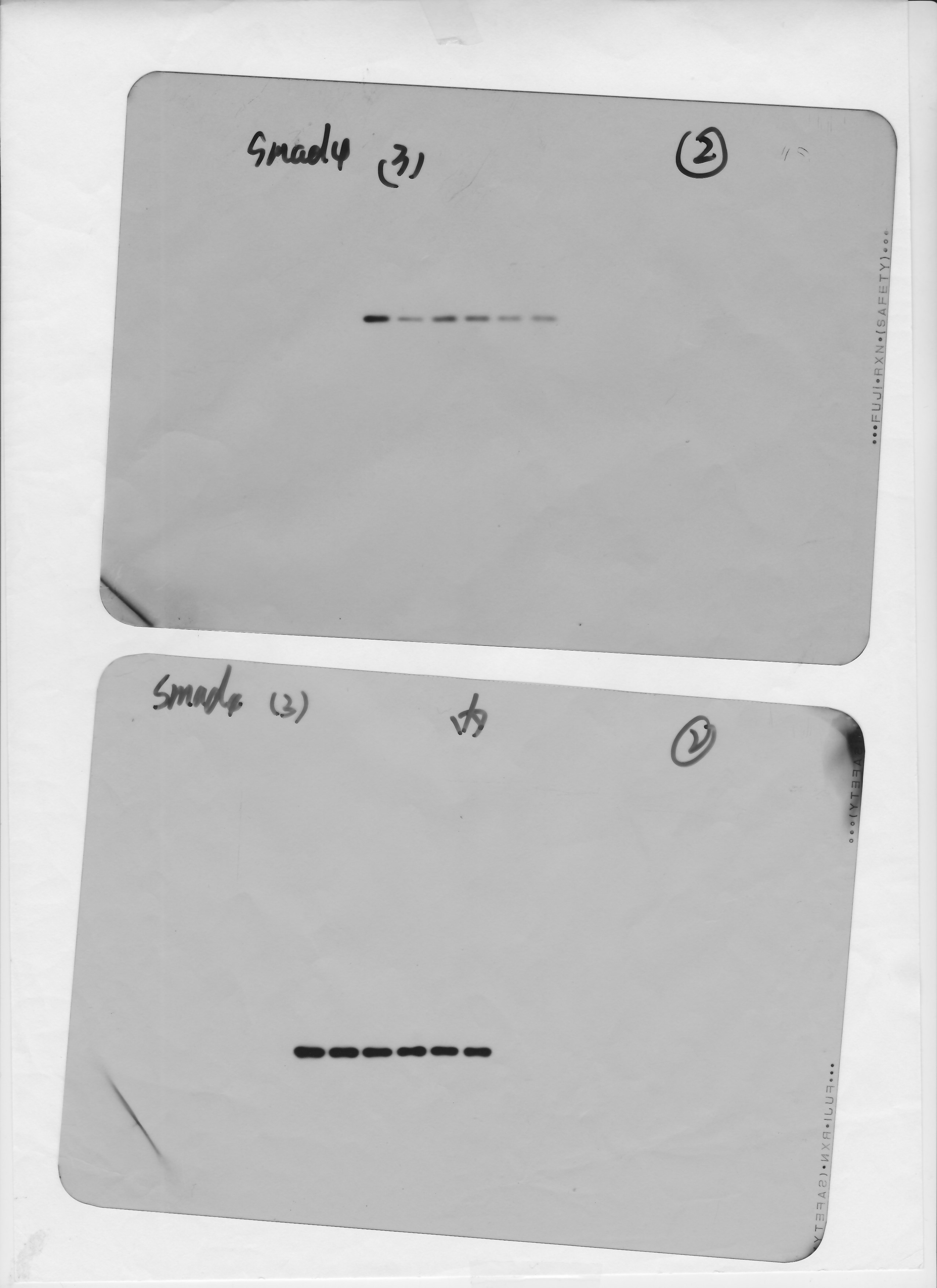

Supplement: Supplementary file 1 — Additional file 1. [file 12872_2020_1646_MOESM1_ESM.zip › 16R3.jpg]

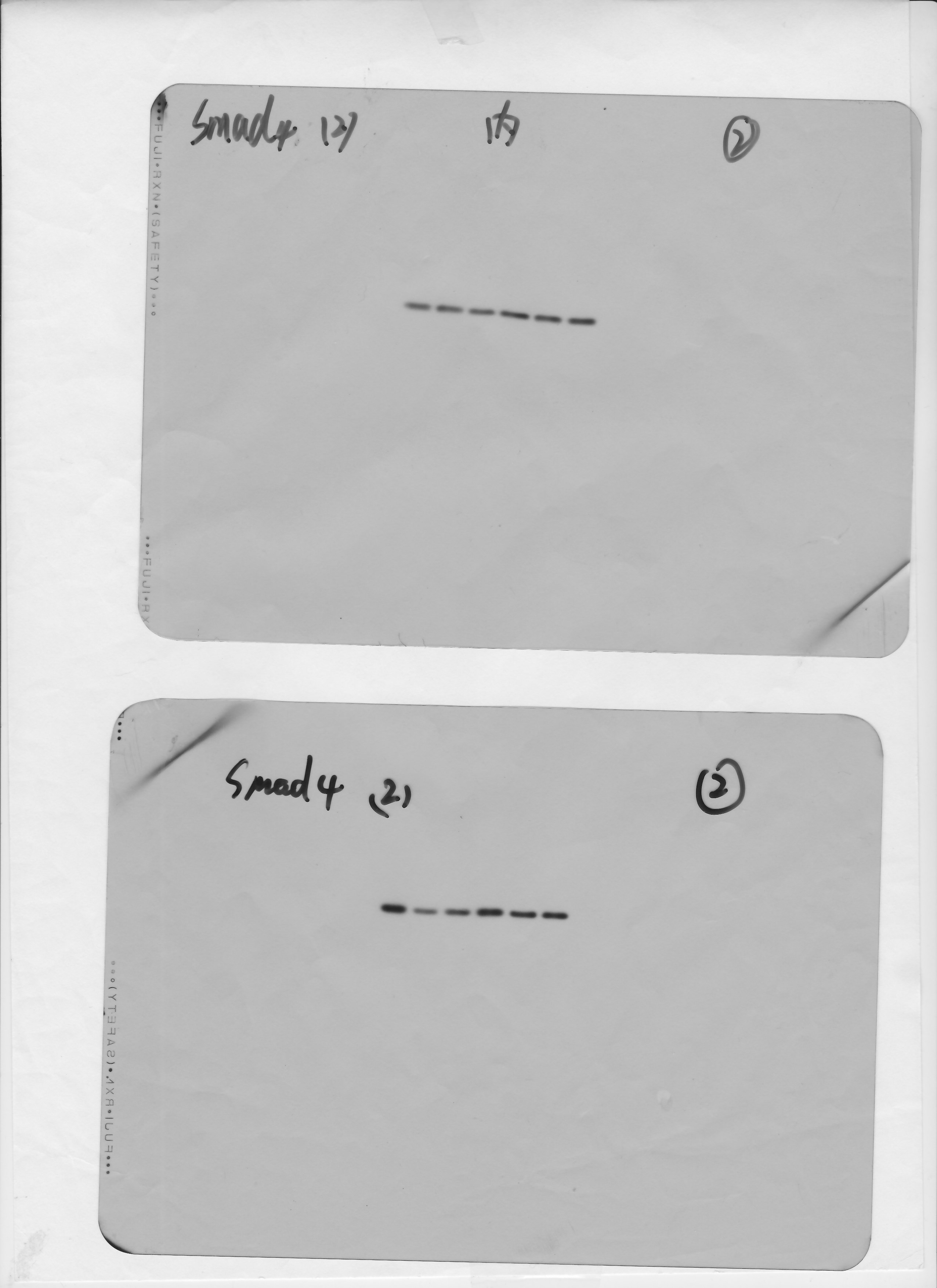

Supplement: Supplementary file 1 — Additional file 1. [file 12872_2020_1646_MOESM1_ESM.zip › 17R3.jpg]

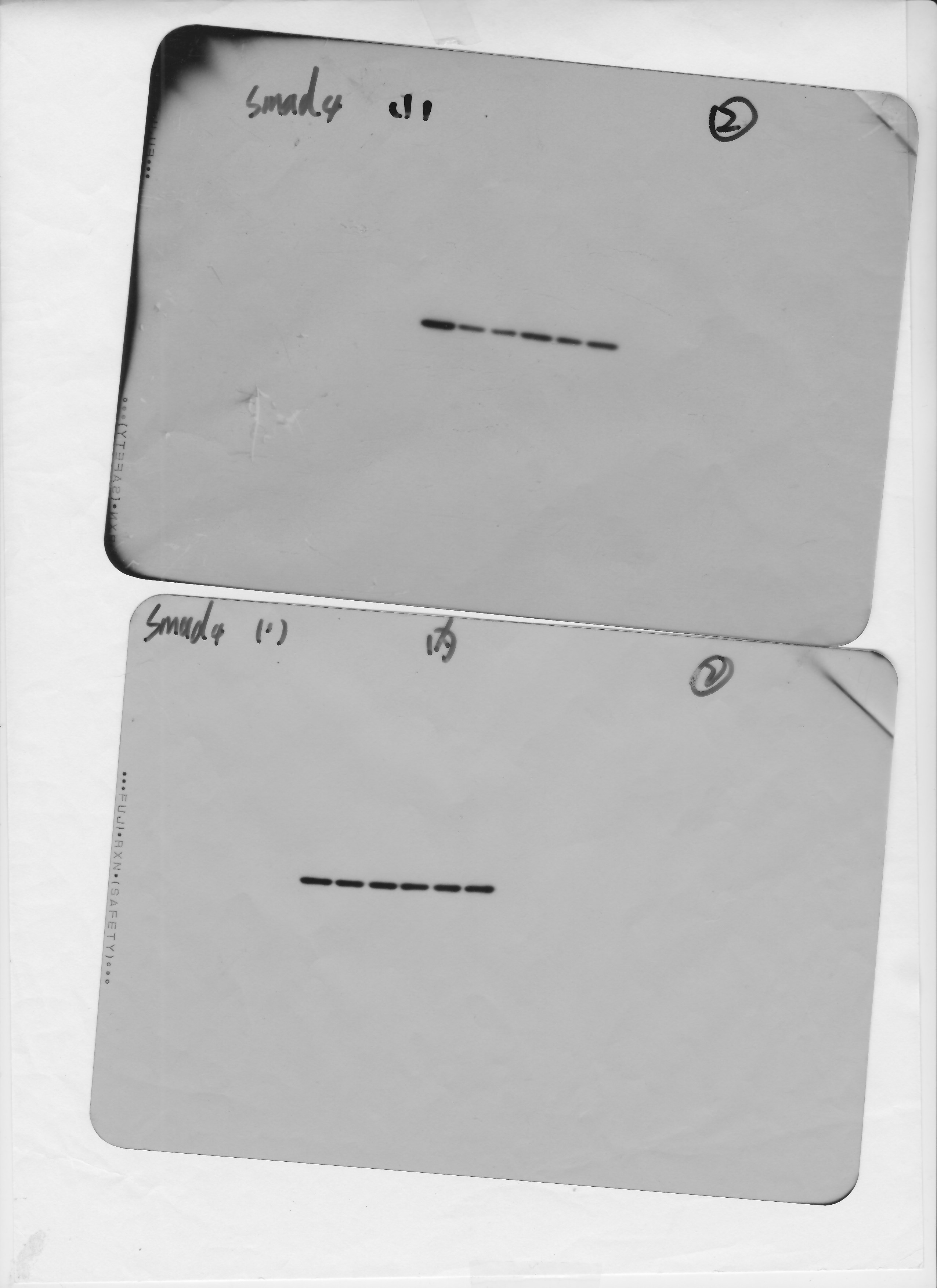

Supplement: Supplementary file 1 — Additional file 1. [file 12872_2020_1646_MOESM1_ESM.zip › 18R3.jpg]

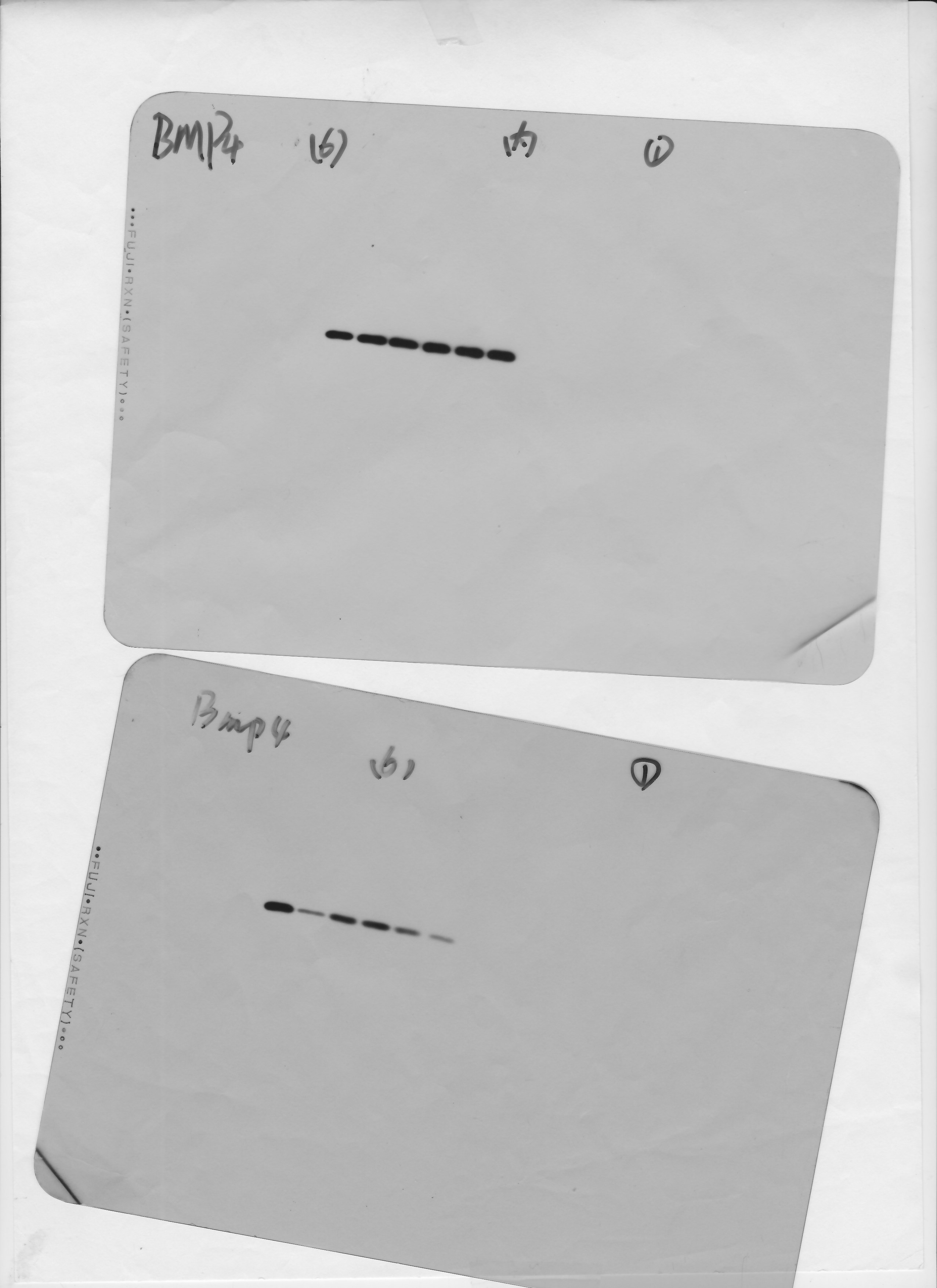

Supplement: Supplementary file 1 — Additional file 1. [file 12872_2020_1646_MOESM1_ESM.zip › 19R3.jpg]

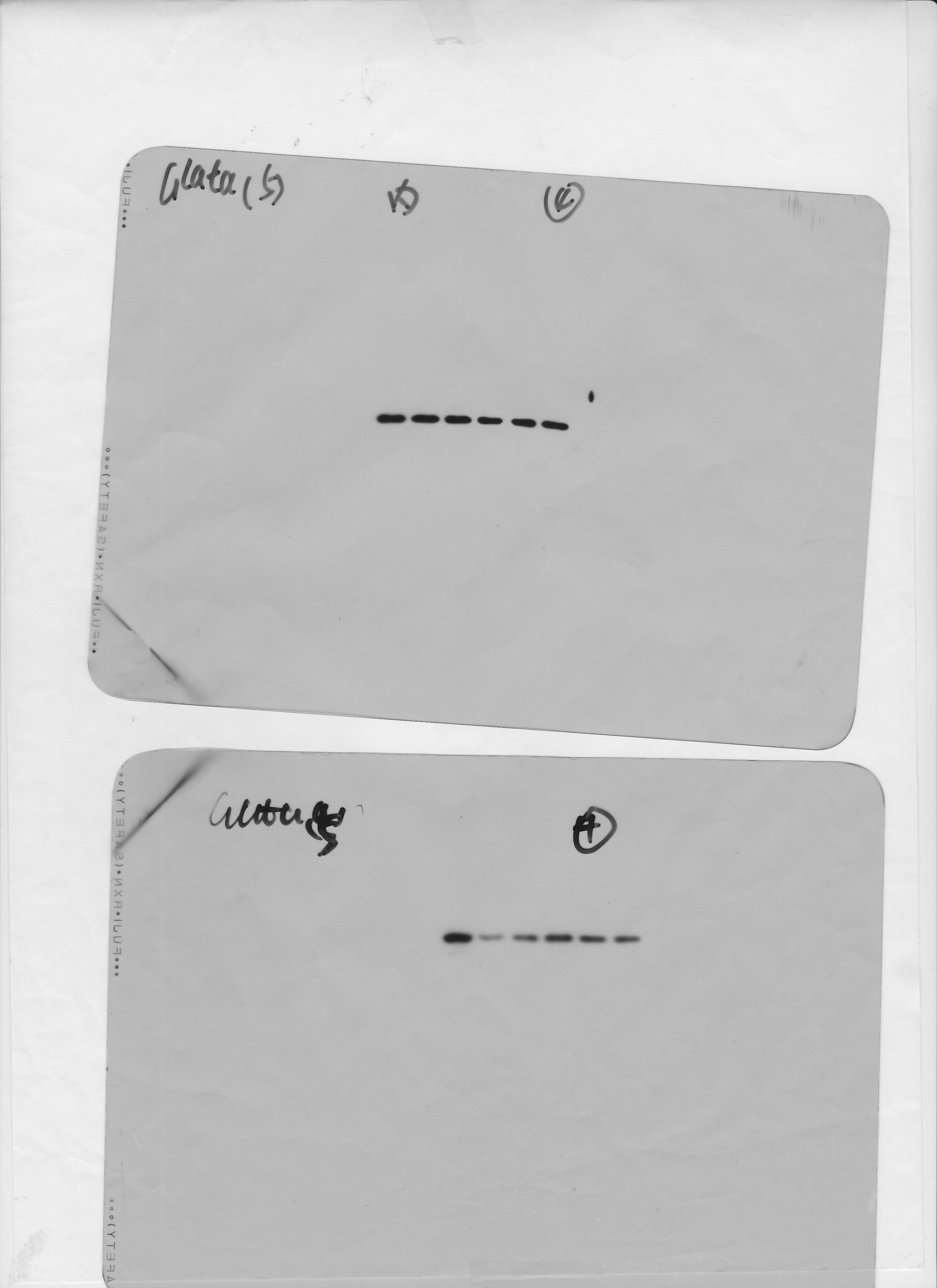

Supplement: Supplementary file 1 — Additional file 1. [file 12872_2020_1646_MOESM1_ESM.zip › 2.jpg]

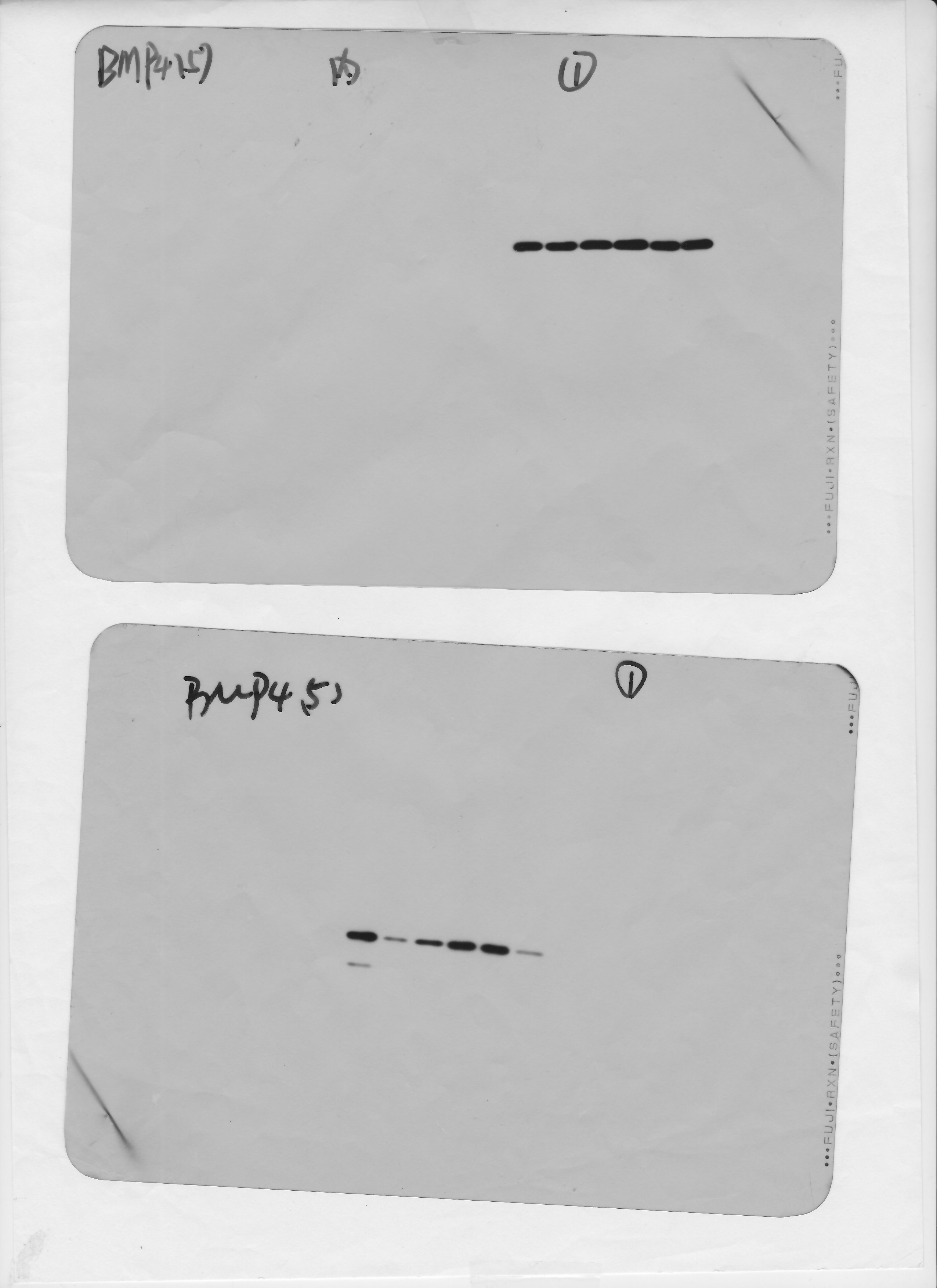

Supplement: Supplementary file 1 — Additional file 1. [file 12872_2020_1646_MOESM1_ESM.zip › 20R3.jpg]

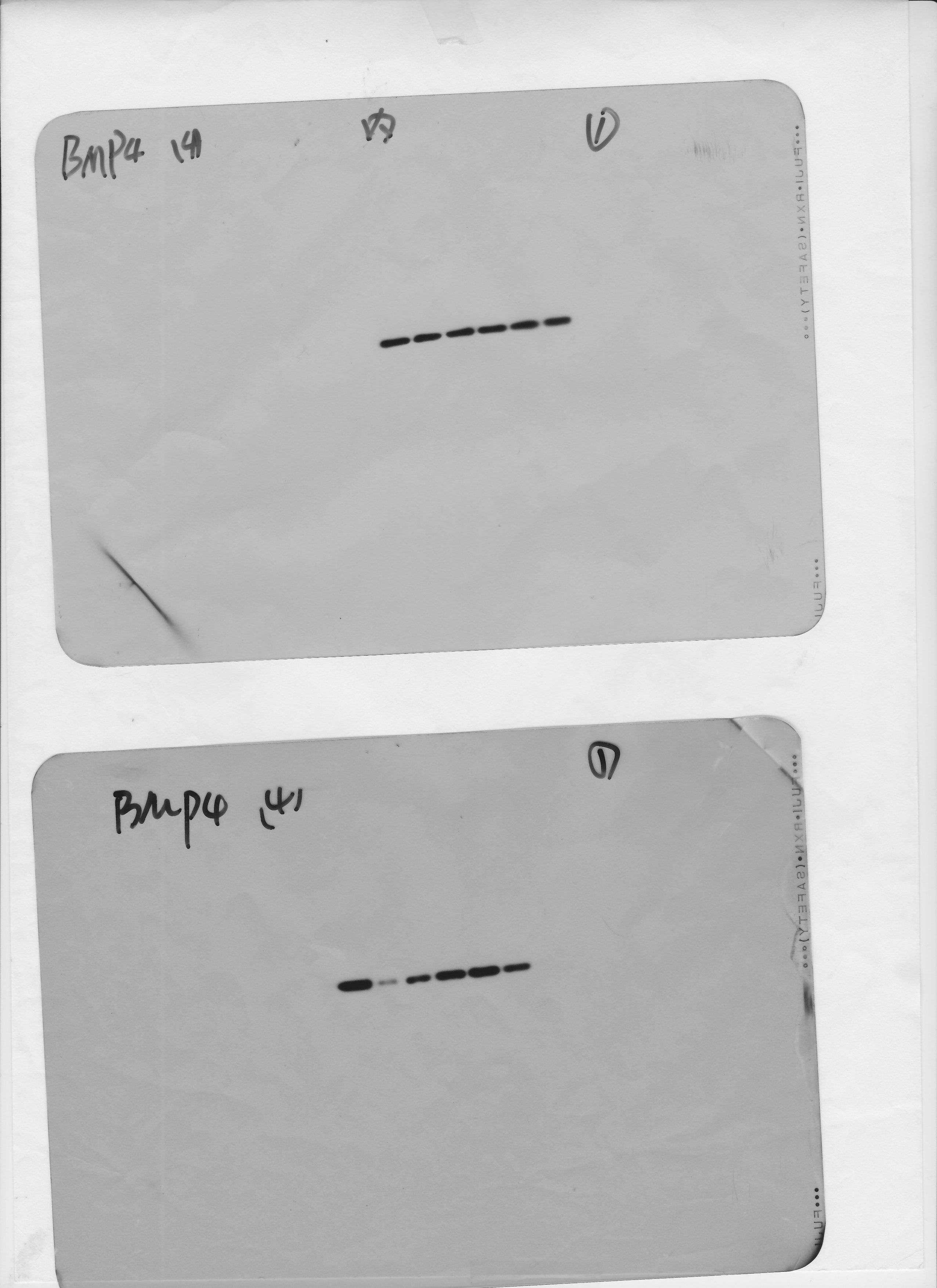

Supplement: Supplementary file 1 — Additional file 1. [file 12872_2020_1646_MOESM1_ESM.zip › 21R3.jpg]

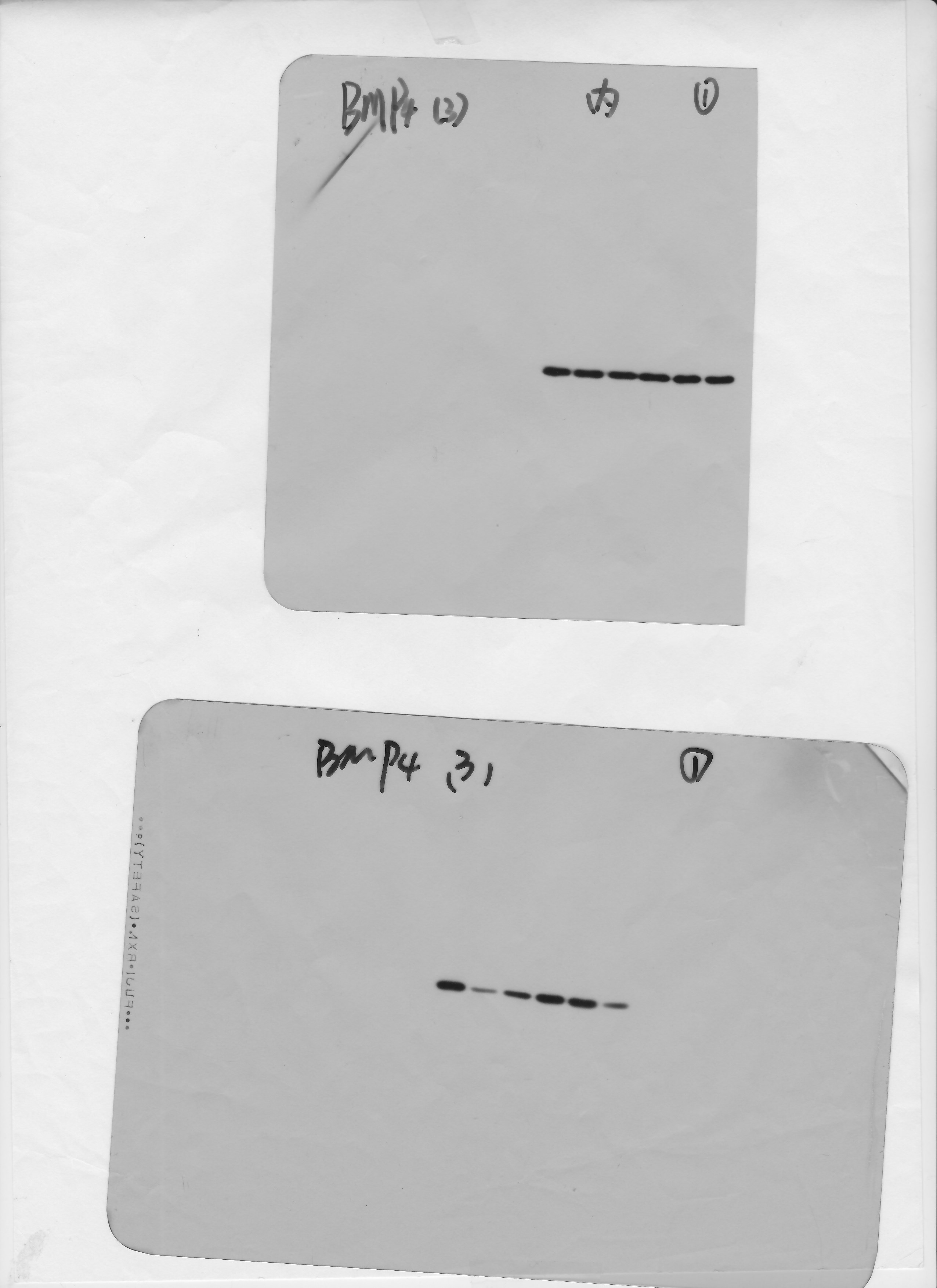

Supplement: Supplementary file 1 — Additional file 1. [file 12872_2020_1646_MOESM1_ESM.zip › 22R3.jpg]

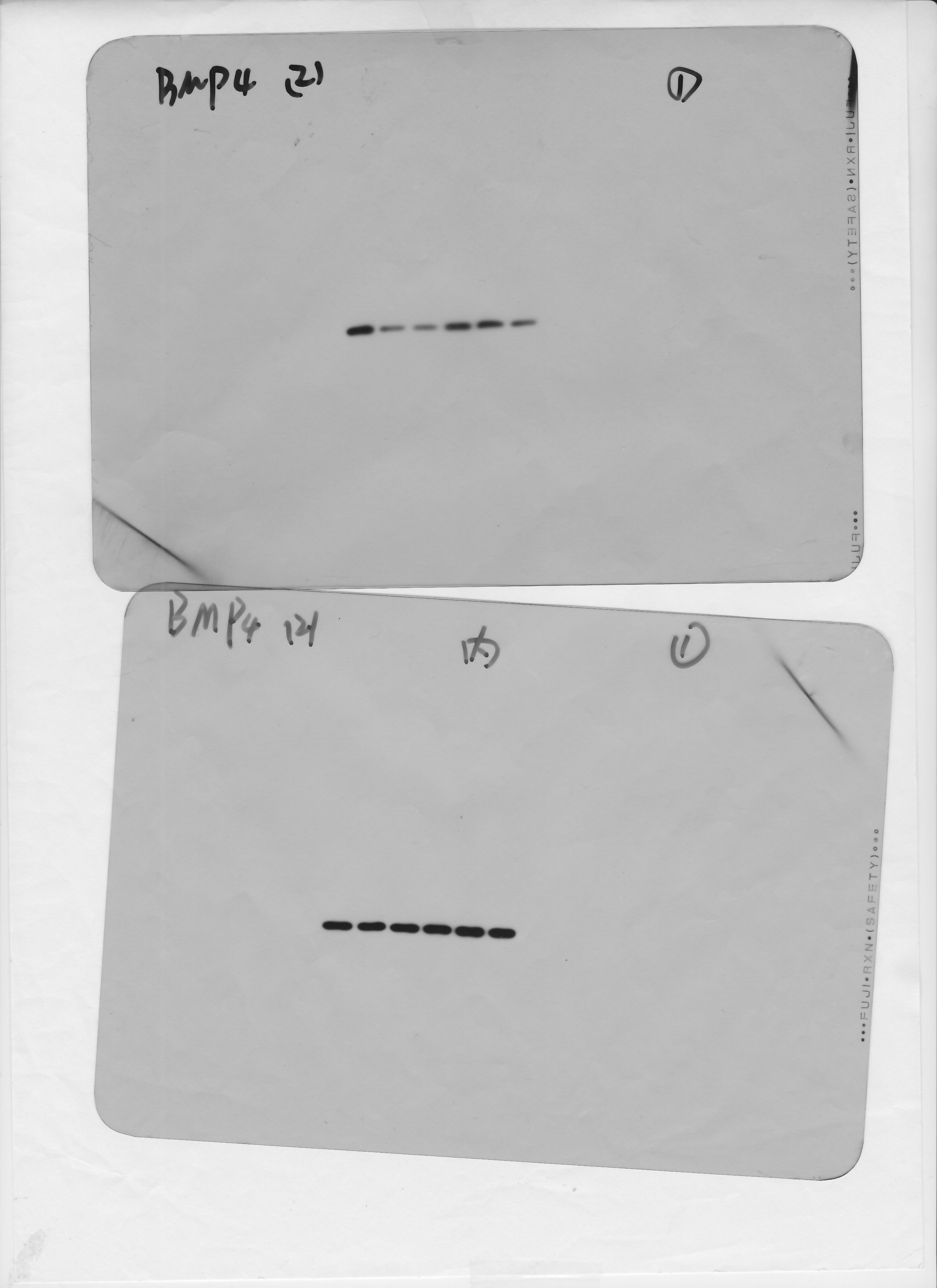

Supplement: Supplementary file 1 — Additional file 1. [file 12872_2020_1646_MOESM1_ESM.zip › 23R3.jpg]

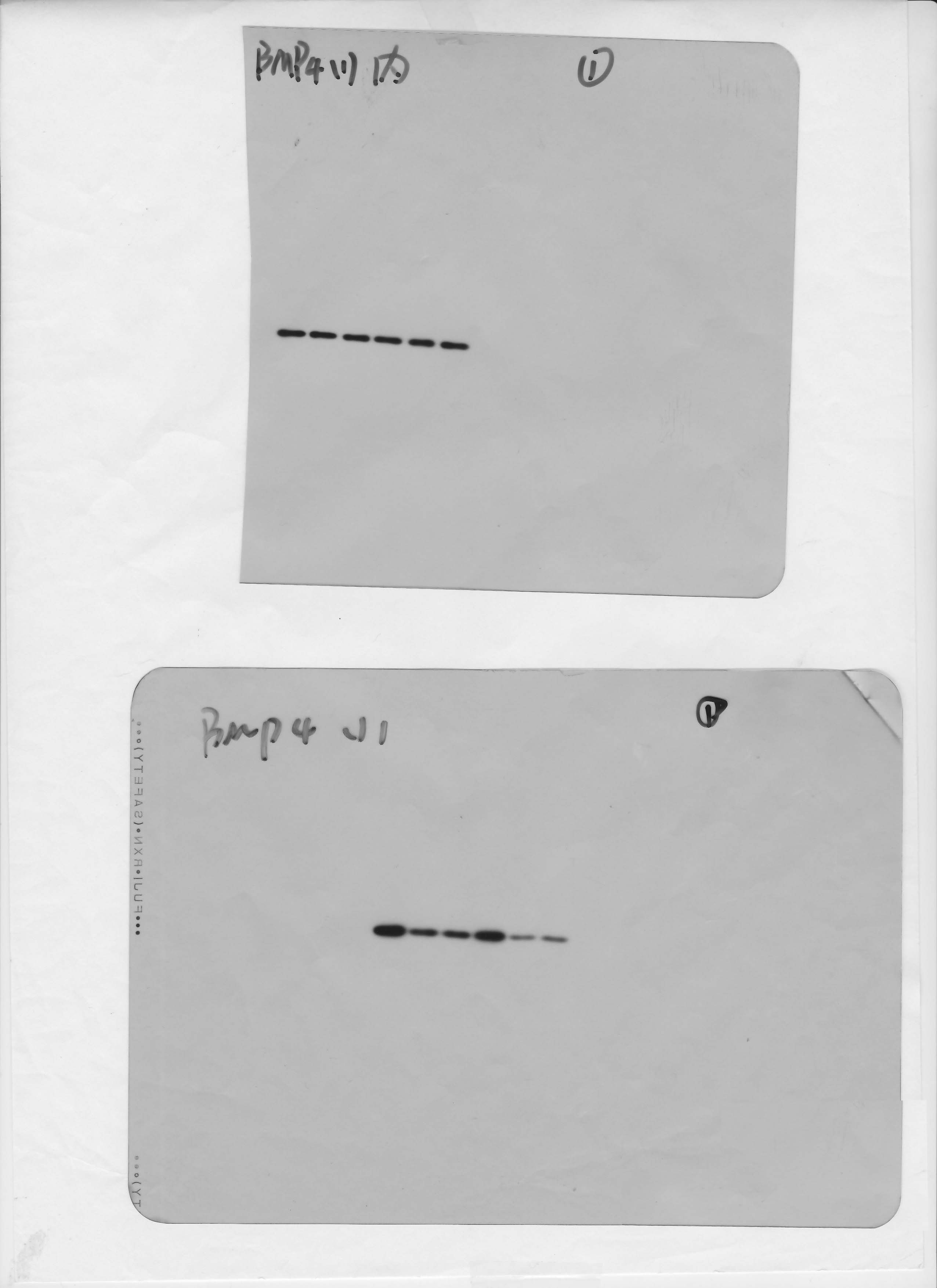

Supplement: Supplementary file 1 — Additional file 1. [file 12872_2020_1646_MOESM1_ESM.zip › 24R3.jpg]

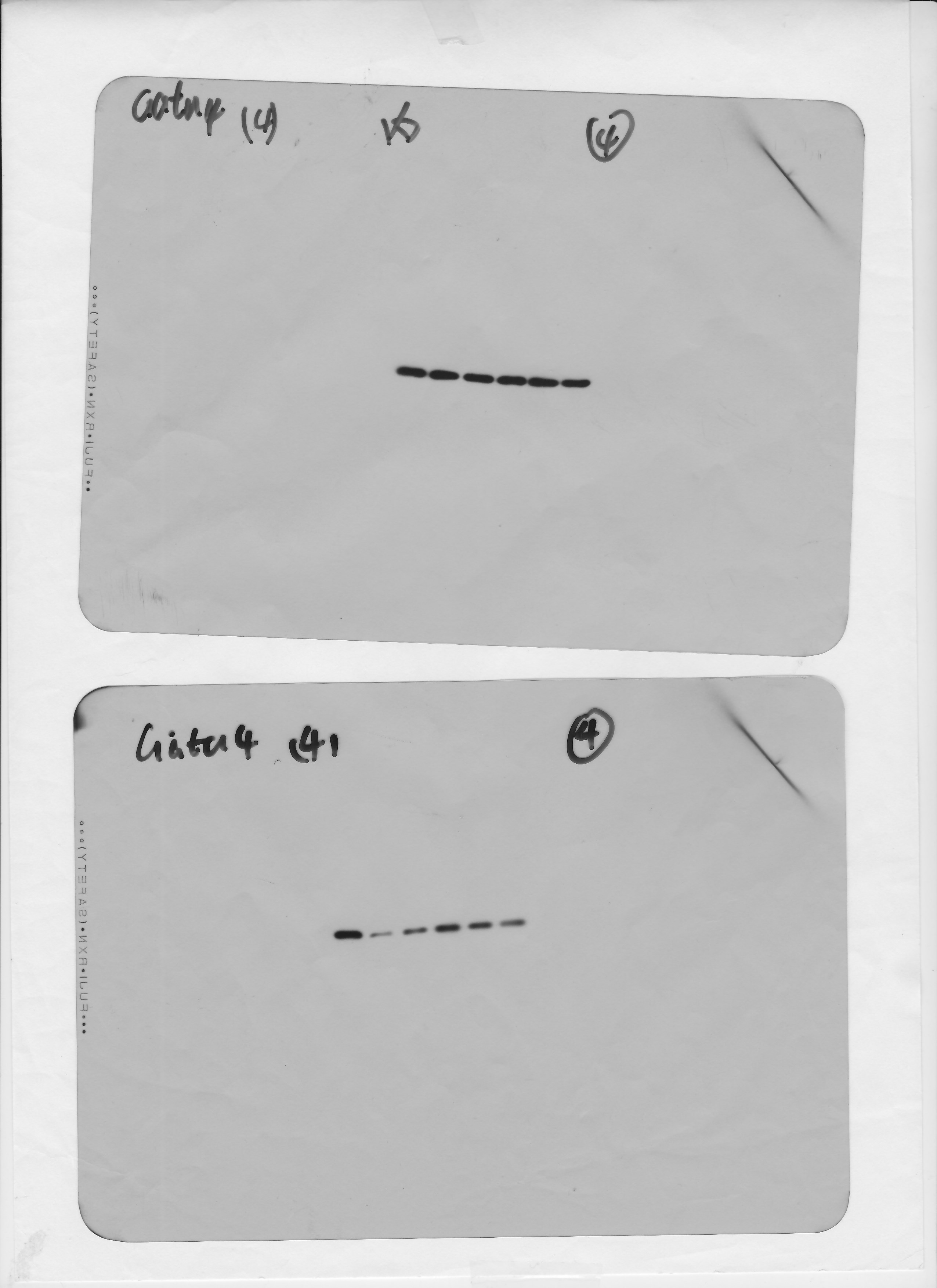

Supplement: Supplementary file 1 — Additional file 1. [file 12872_2020_1646_MOESM1_ESM.zip › 3.jpg]

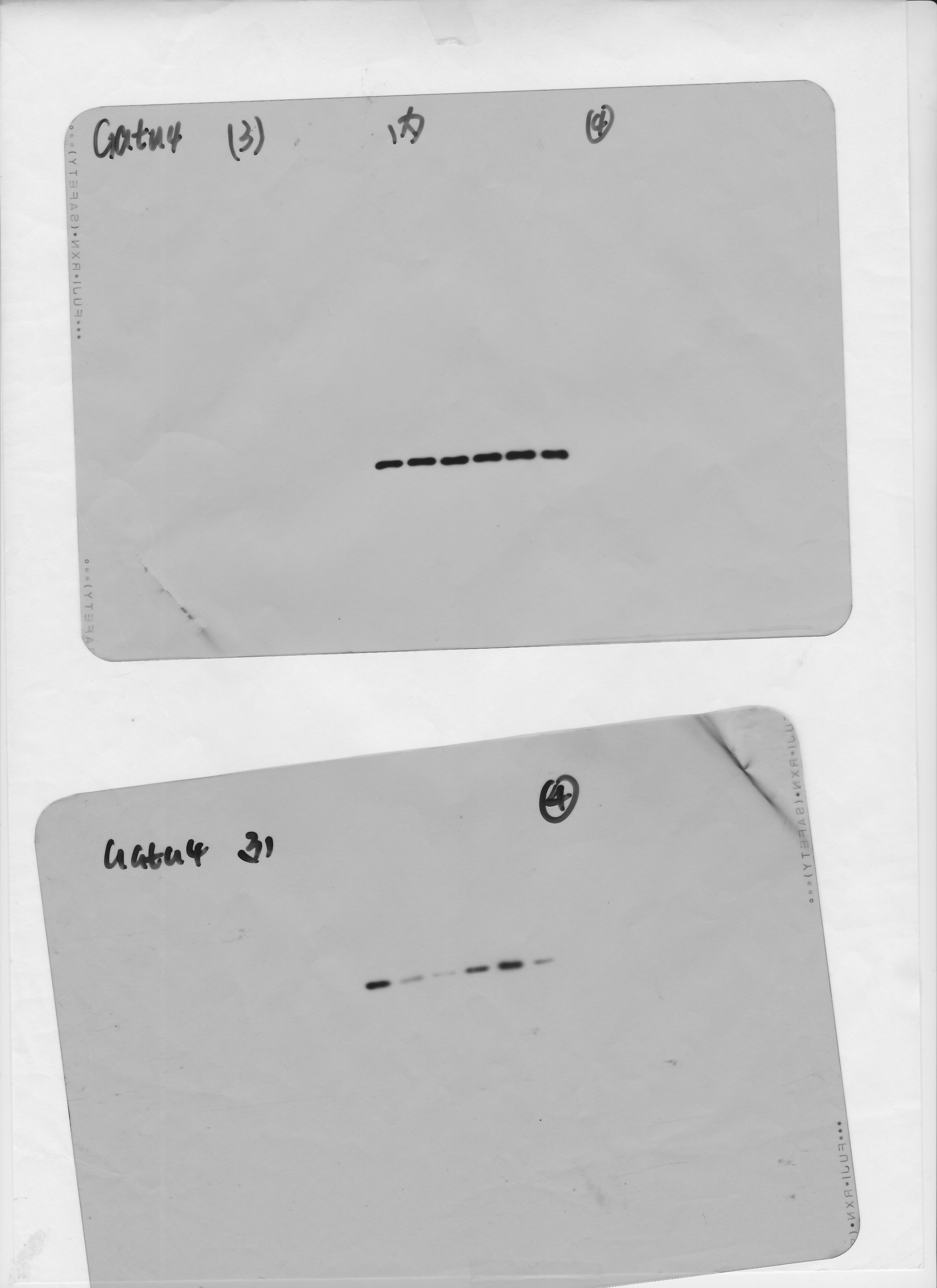

Supplement: Supplementary file 1 — Additional file 1. [file 12872_2020_1646_MOESM1_ESM.zip › 4.jpg]

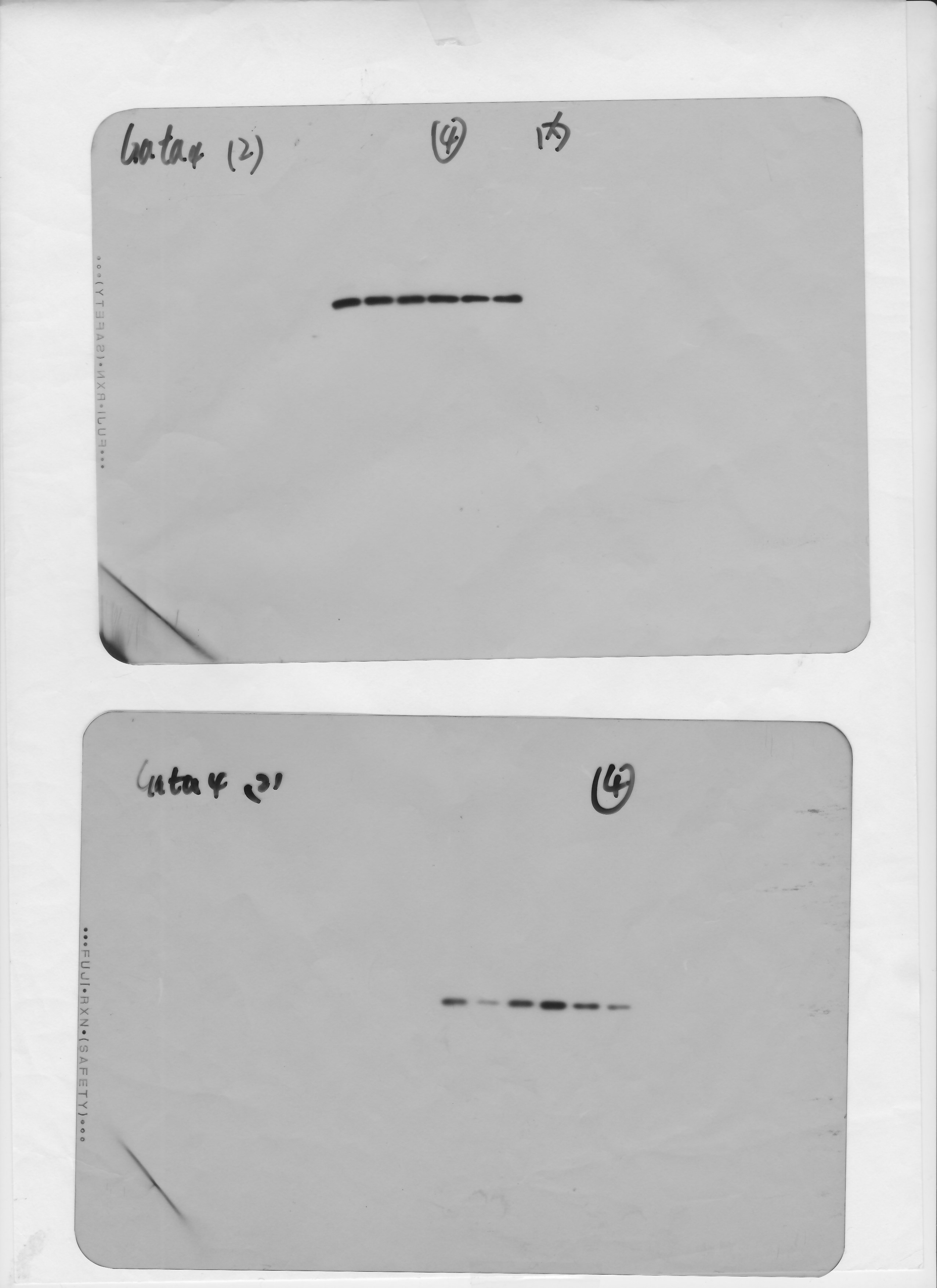

Supplement: Supplementary file 1 — Additional file 1. [file 12872_2020_1646_MOESM1_ESM.zip › 5.jpg]

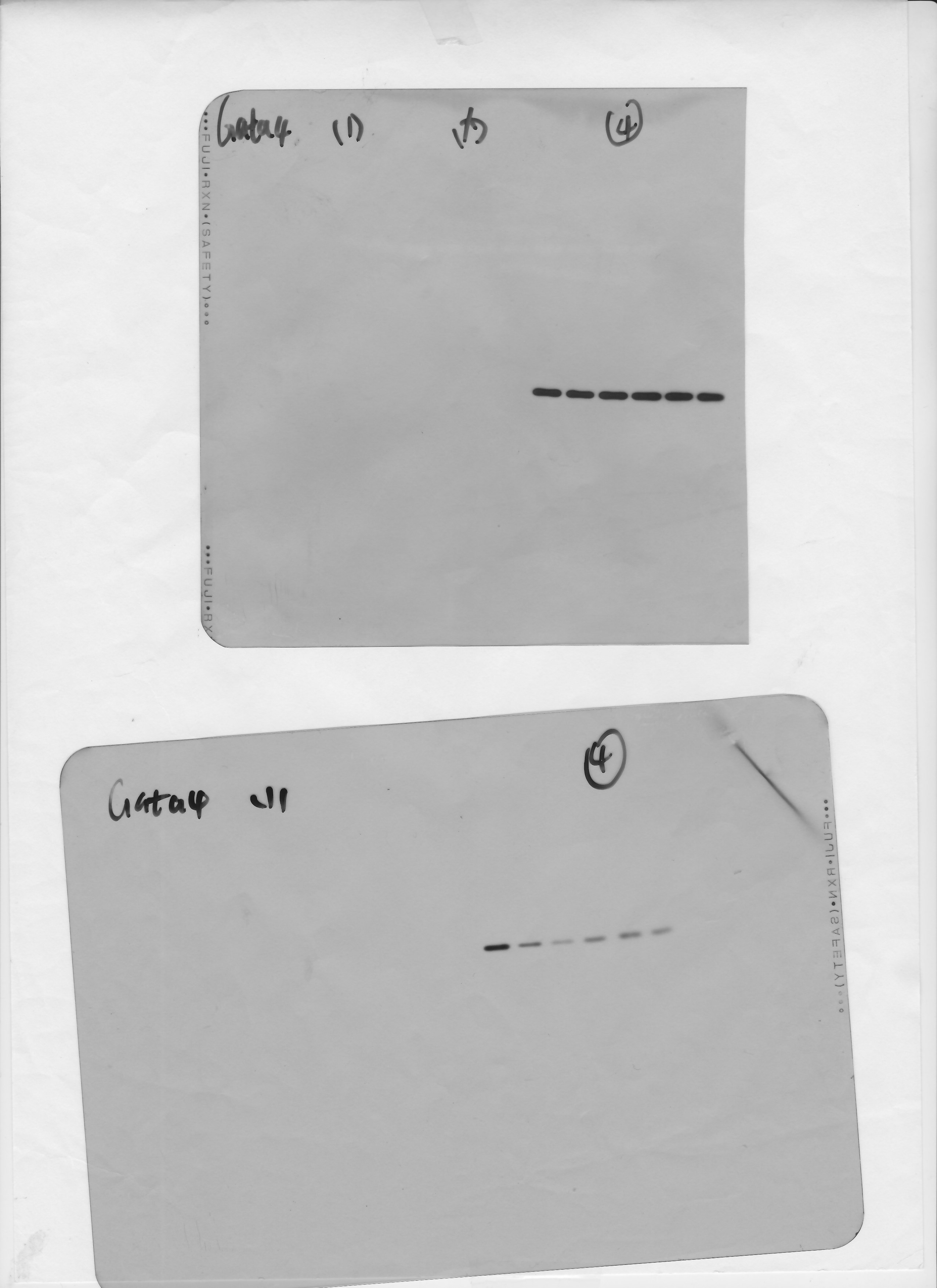

Supplement: Supplementary file 1 — Additional file 1. [file 12872_2020_1646_MOESM1_ESM.zip › 6.jpg]

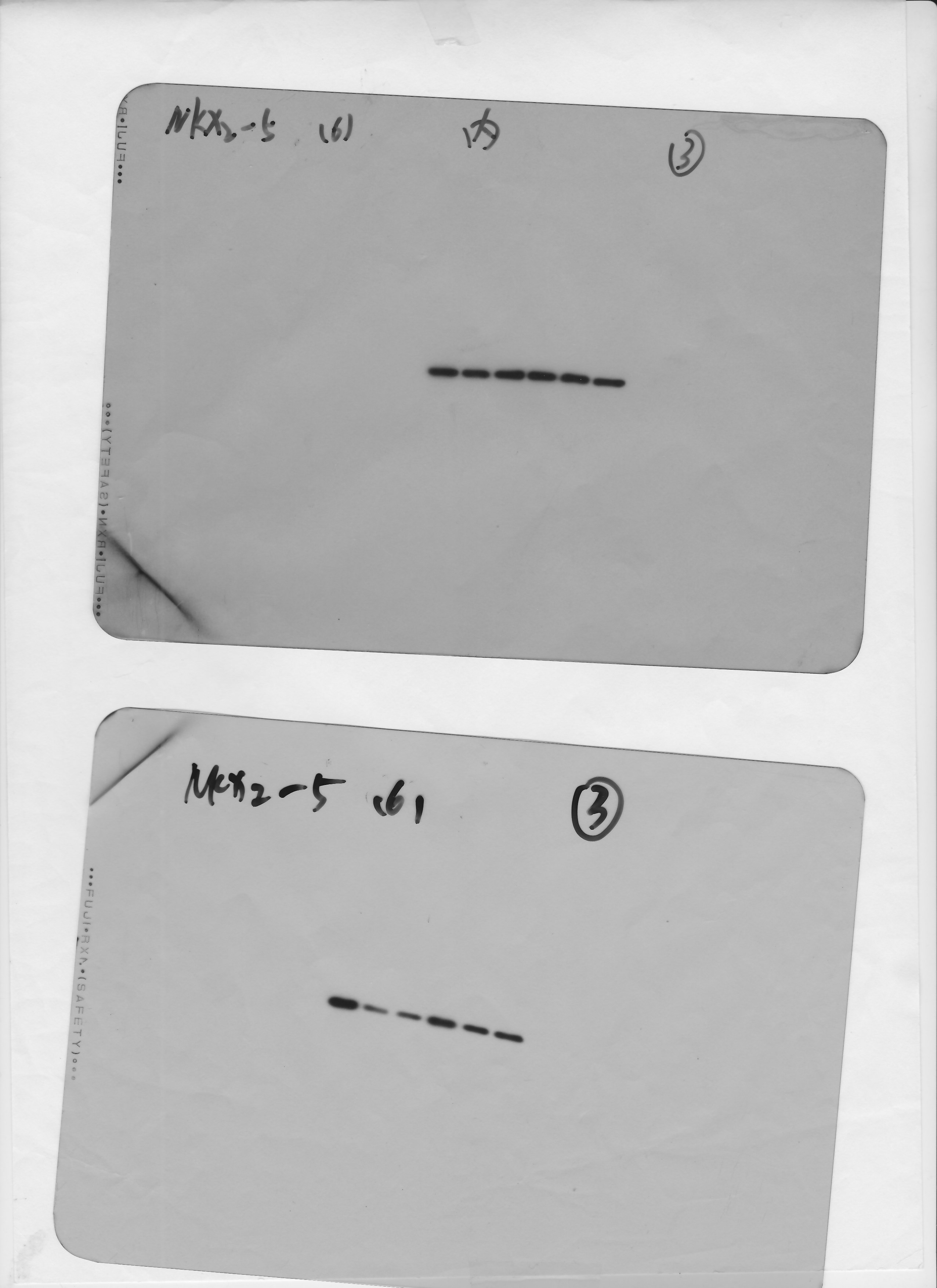

Supplement: Supplementary file 1 — Additional file 1. [file 12872_2020_1646_MOESM1_ESM.zip › 7.jpg]

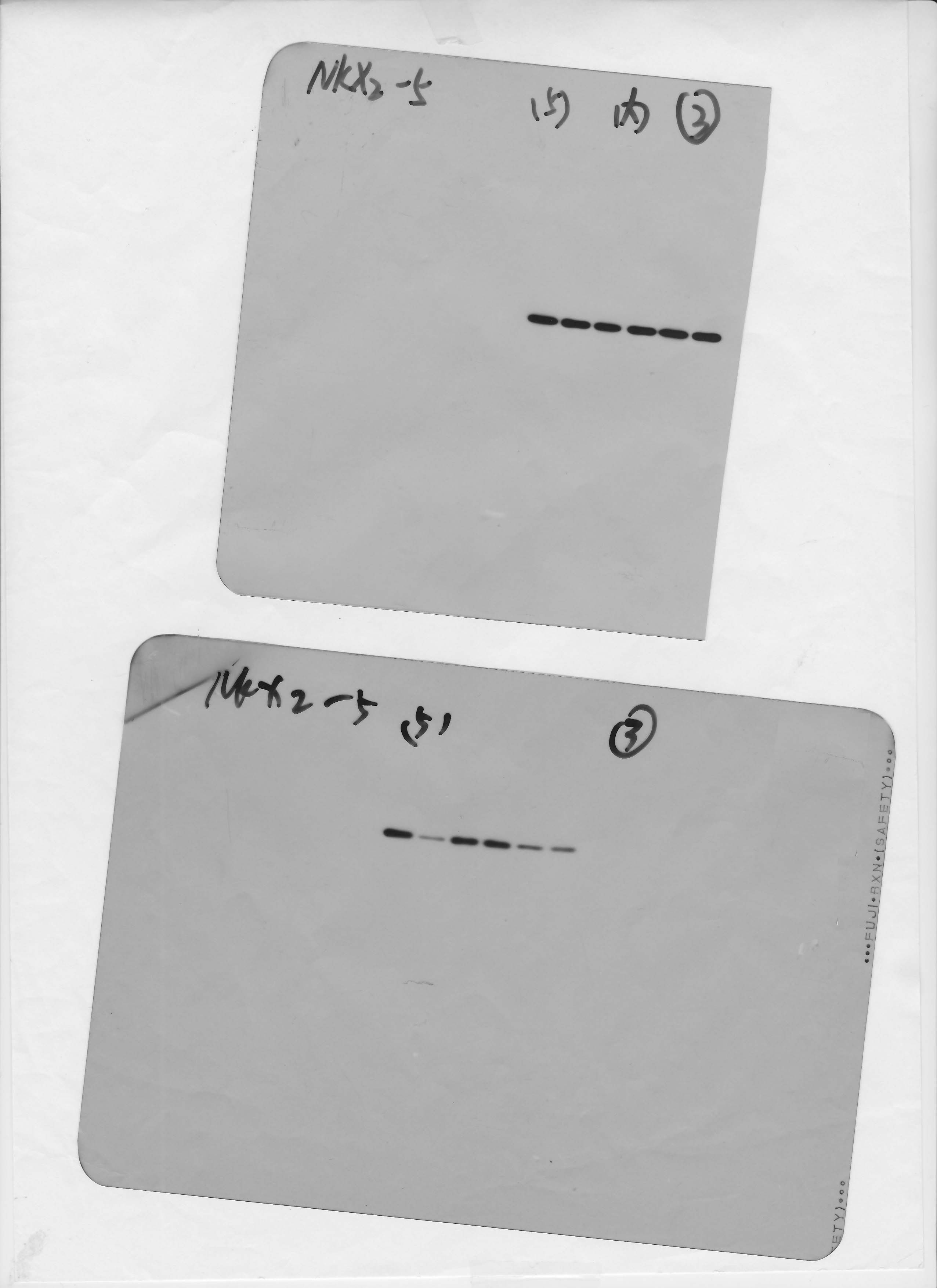

Supplement: Supplementary file 1 — Additional file 1. [file 12872_2020_1646_MOESM1_ESM.zip › 8.jpg]

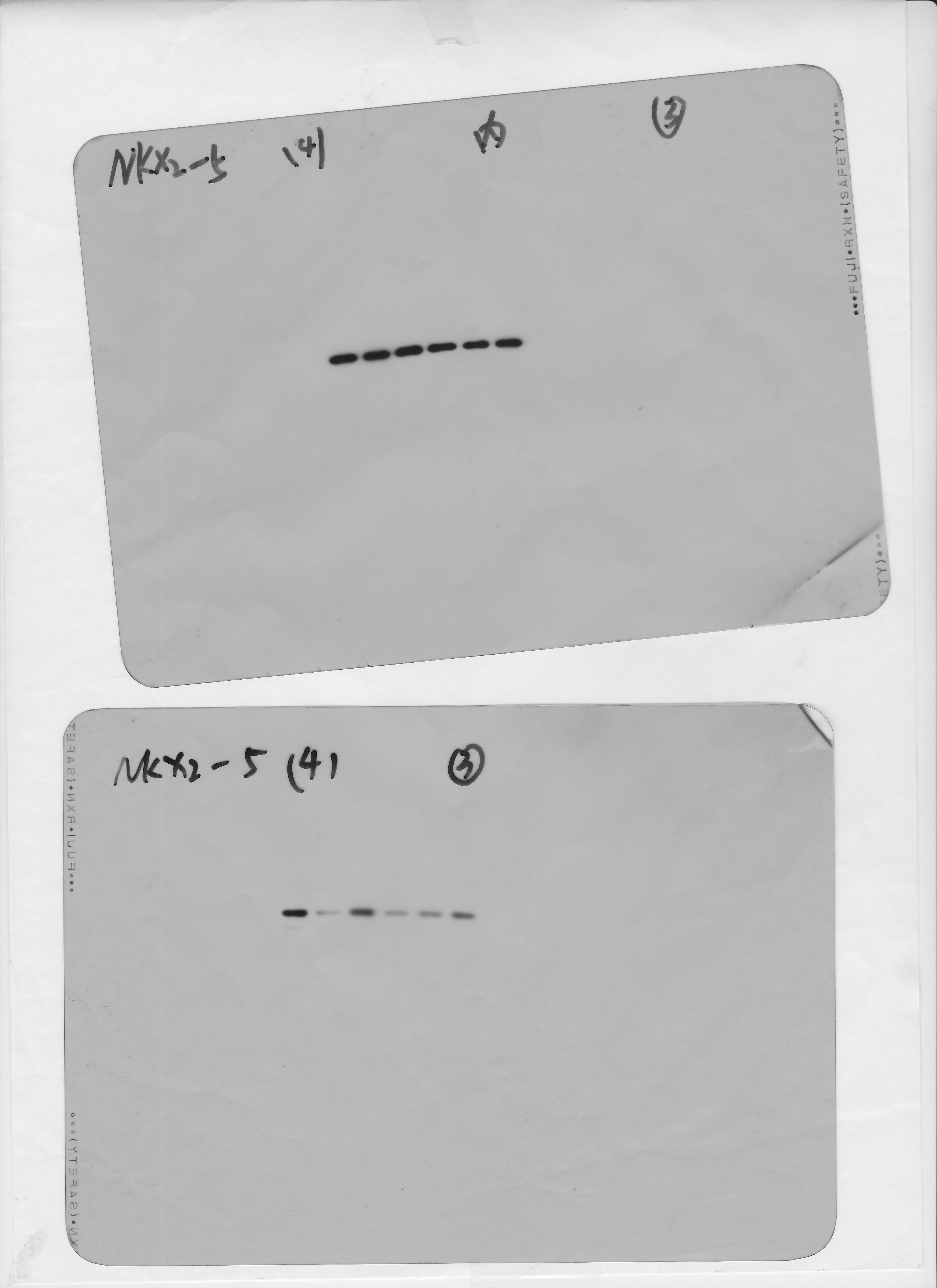

Supplement: Supplementary file 1 — Additional file 1. [file 12872_2020_1646_MOESM1_ESM.zip › 9.jpg]
